# Broad rim lesions are a new pathological and imaging biomarker for rapid disease progression in multiple sclerosis

**DOI:** 10.1038/s41591-025-03625-7

**Published:** 2025-04-29

**Authors:** Luisa Klotz, Joost Smolders, Jussi Lehto, Markus Matilainen, Lukas Lütje, Luzia Buchholz, Stefanie Albrecht, Carolin Walter, Julian Varghese, Heinz Wiendl, Marjo Nylund, Christian Thomas, Maria Gardberg, Aletta M. R. van den Bosch, Laura Airas, Inge Huitinga, Tanja Kuhlmann

**Affiliations:** 1 Department of Neurology, University Hospital Münster, Münster, Germany; 2 Neuroimmunology Research Group, Netherlands Institute for Neuroscience, Amsterdam, the Netherlands; 3 MS Center ErasMS, Departments of Neurology and Immunology, Erasmus MC, University Medical Center Rotterdam, Rotterdam, the Netherlands; 4 Turku PET Centre, Turku University Hospital, Turku, Finland; 5 Neurocenter, Turku University Hospital, Turku, Finland; 6 InFLAMES Research Flagship, University of Turku, Turku, Finland; 7 Faculty of Science and Engineering, Åbo Akademi University, Turku, Finland; 8 Clinical Neurosciences, University of Turku, Turku, Finland; 9 Institute of Neuropathology, University Hospital Münster, Münster, Germany; 10 Institute of Medical Informatics, University Hospital Münster, Münster, Germany; 11 Department of Neurology and Neurophysiology, Medical Center, University of Freiburg, Freiburg, Germany; 12 Department of Pathology, Turku University Hospital and Institute of Biomedicine, University of Turku, Turku, Finland; 13 Swammerdam Institute for Life Sciences, University of Amsterdam, Amsterdam, the Netherlands

**Keywords:** Neuroimmunology, Multiple sclerosis

## Abstract

Current multiple sclerosis (MS) treatments reduce relapse activity but have limited impact on disease progression. Clinical trials targeting progression often fail because of insufficient understanding of its underlying mechanisms. This study analyzed a clinically well-characterized MS autopsy cohort from the Netherland Brain Bank (186 individuals) from which we selected donors exhibiting opposite disease trajectories of slow versus rapid progression. We performed extensive unbiased histology and spatial transcriptomics, which unveiled a distinct MS lesion type marked by an extensive myeloid cell rim with cellular and transcriptional signatures of innate immune activation, inflammatory cytokine production, unfolded protein response and apoptosis. Presence of this particular lesion type was linked to rapid disease progression. An independent translocator protein 18-kDa positron emission tomography study (114 individuals) validates the association between lesions with a broad myeloid cell rim and disease progression in individuals with MS. Our findings offer crucial insights into the mechanisms behind MS progression, identifying broad rim lesions as a biomarker for rapid disease progression and potentially guiding patient selection for future therapeutic trials targeting central nervous system intrinsic inflammation.

## Abstract (web summary)

This study identifies a unique multiple sclerosis lesion, the broad rim lesion, which is associated with rapid disease progression and marked by a distinct inflammatory profile involving innate inflammation, cellular stress, unfolded protein response and apoptosis.

## Main

Multiple sclerosis (MS) is the most prevalent inflammatory demyelinating disease of the central nervous system (CNS), affecting 2.8 million people worldwide^1^. The disease course varies, with disability accumulation driven by both relapse-related activity and relapse-independent progression^2^. Traditionally, MS has been classified into relapsing-remitting MS, secondary progressive MS and primary progressive MS. However, this concept has been challenged by evidence of relapses during progressive phases and the early occurrence of progression independent of relapses in relapsing MS^3^. These findings support a continuum of MS driven by distinct pathophysiologies^3–5^. Relapse activity is linked to peripheral immune cell activation and CNS infiltration, leading to focal inflammation and lesion formation, as evidenced by the efficacy of immunomodulating drugs primarily targeting lymphocyte activation, proliferation or transmigration^6,7^. However, these treatments have limited effects on relapse-independent progression, suggesting that distinct CNS mechanisms, including persistent focal and non-focal inflammation and failure of compensatory processes like plasticity and remyelination, contribute to progression independent of relapses^8^.

We clinically characterized a large cohort of donors with MS from the Netherlands Brain Bank (NBB), stringently selecting patients with either very rapid disease progression resulting in substantial disability accumulation already a few years after disease onset (MSwRP), or very slow disease progression where relevant disability milestones were reached after decades (MSwSP). These two extremes of the MS continuum were analyzed for lesion pathology using histology and spatial transcriptomics; findings were correlated with imaging studies.

## Results

### Clinical characteristics of histology cohort

**Fig. 1:**
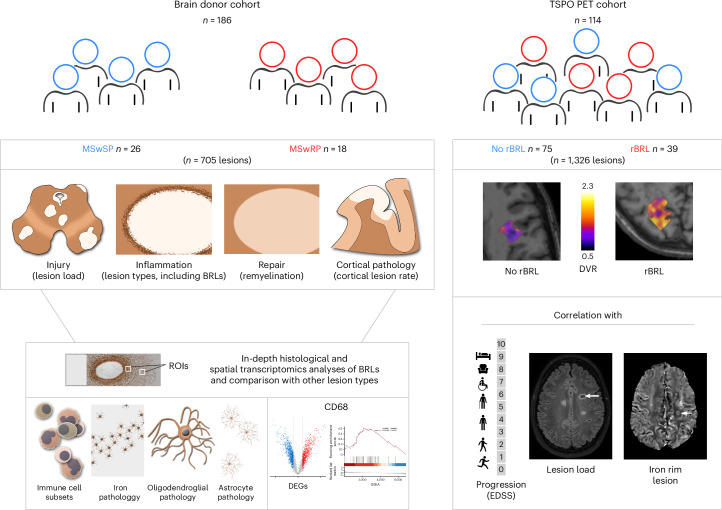
Schematic illustration of the study design. Left, the brain donor cohort from the NBB included 186 donors. We selected 44 patients with two extreme phenotypes, 26 patients with a very slow disease progression (MSwSP) and 18 patients with a very rapid disease progression (MSwRP), to perform comprehensive histological analyses of a total of 705 MS lesions. BRLs were further characterized using in-depth histological and spatial transcriptomics. Right, the TSPO PET imaging cohort included *n* = 114 patients with MS with 1,326 lesions in total. Among the 114 patients, 39 patients displayed radiological BRLs (rBRLs). Presence of rBRL was correlated with additional imaging findings and disease progression. ROI, region of interest. DEG, differentially expressed gene. GSEA, gene set enrichment analysis.

**Fig. 2:**
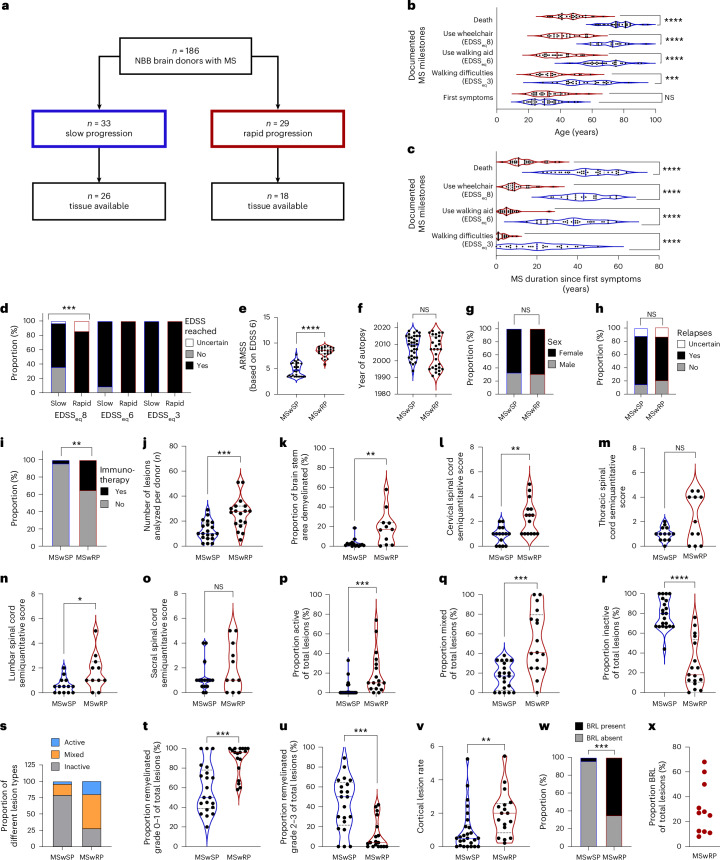
Patients in the MSwRP group have similar EDSS scores at death compared to patients in the MSwSP group but display more severe histopathological characteristics. **a**, Stratification of the patient cohort. **b**,**c**, Individuals in the MSwRP group reached equivalents of clinical milestones at an earlier age (**b**) and after a shorter disease duration (**c**) compared to individuals in the MSwSP group. In the violin plots, *P* values were determined using Welch’s analysis of variance (ANOVA), Bonferroni-corrected for multiple testing. **d**, A higher proportion of individuals in the MSwRP group reached an EDSS of 8 compared to individuals in the MSwSP group; *P* values were determined using a Fisher’s exact test. **e**, Individuals in the MSwRP group reached significantly higher scores using ARMSS; the two-tailed *P* value was determined using a Mann–Whitney *U*-test. **f**–**h**, There was no difference between the MSwSP and MSwRP groups regarding the year of autopsy (**f**), the sex ratio (**g**) and the proportion of patients with relapses (**h**). **f**, Two-tailed *P* values were determined using a Mann–Whitney *U*-test. **g**,**h**, *P* values were determined using a Fisher’s exact test. **i**, A higher proportion of patients with rapid disease progression received immunotherapy; *P* values were determined using a Fisher’s exact test. **j**–**v**, The MSwRP group had a higher number of lesions (**j**), a higher proportion of demyelinated brain stem area (**k**), a higher cervical lesion load (**l**), no increase in thoracic lesion load (**m**), a higher lumbar lesion load (**n**), no increase in sacral lesion load (**o**), a higher proportion of active (**p**,**s**) and mixed (**q**,**s**) lesions, a lower proportion of inactive lesions (**r**,**s**), a higher proportion of lesions with limited remyelination (**t**), a lower proportion of lesions with marked remyelination (**u**) and a higher cortical lesion rate (**v**). In the violin plots, two-tailed *P* values were determined using a Mann–Whitney *U*-test. **w**, Individuals in the MSwRP group had a significantly higher proportion of BRLs; the *P* value was determined using a Fisher’s exact test. **x**, BRLs consisted of 8–68% of total lesions in the MSwRP group. *P ≤ 0.05; **P ≤ 0.01; ***P ≤ 0.001; ****P < 0.0001; NS, not significant. Source data

We evaluated the disease course of 186 donor files from the NBB with autopsy material by two independent experts (Methods and Fig. 1). Patients at the extremes of the disease spectrum were identified, showing either very rapid (MSwRP, *n* = 29) or very slow progression (MSwSP, *n* = 33) (Fig. 2a). Both groups had comparable ages at onset, but the MSwRP group reached clinical milestones significantly earlier and after a shorter disease duration than the MSwSP group (Fig. 2b,c). All the MSwRP group and most of the MSwSP group reached an Expanded Disability Status Scale (EDSS) score of 8 (Fig. 2d). Comparing disability, the MSwRP group had significantly higher age-adjusted multiple sclerosis severity scores (ARMSS) than the MSwSP group (Fig. 2e)^9^. The year of the autopsy did not differ significantly, excluding temporal changes in disease course (Fig. 2f). Both patient groups had similar sex distributions (68% versus 67% females) and a similar proportion of relapsing disease courses (71% versus 78%) (Fig. 2g,h), but more patients in the MSwRP group received immunotherapy (35% versus 6%) (Fig. 2i).

### Histological features of MSwRP

To identify the histopathological characteristics linked to rapid disease progression, we compared the two patient cohorts in detail. Histological analysis was conducted on stained sections or scanned images from 18 of 29 MSwRP and 26 of 33 MSwSP brain donors (Fig. 1; clinical details are shown in Supplementary Table 3). The MSwRP group exhibited a higher number of lesions per patient (Fig. 2j) and a higher lesion load in the brain stem, cervical and lumbar spinal cord, with similar trends in the thoracic and sacral spinal cord (Fig. 2k–o). Lesion composition was classified as active, mixed active and inactive (mixed) or inactive based on myeloid cell density and location^10^. The MSwRP group showed higher proportions of active and mixed lesions and fewer inactive lesions (Fig. 2p–s). Remyelination was assessed with a semiquantitative score from 0 (no remyelination) to 3 (complete remyelination of the lesion area)^11^; the MSwSP group had more lesions with marked remyelination (score of 2 or 3) (Fig. 2t,u). Additionally, the MSwRP group displayed a higher cortical lesion rate (that is, the average number of cortical lesions per tissue block per patient) compared to the MSwSP group (Fig. 2v).

**Fig. 3:**
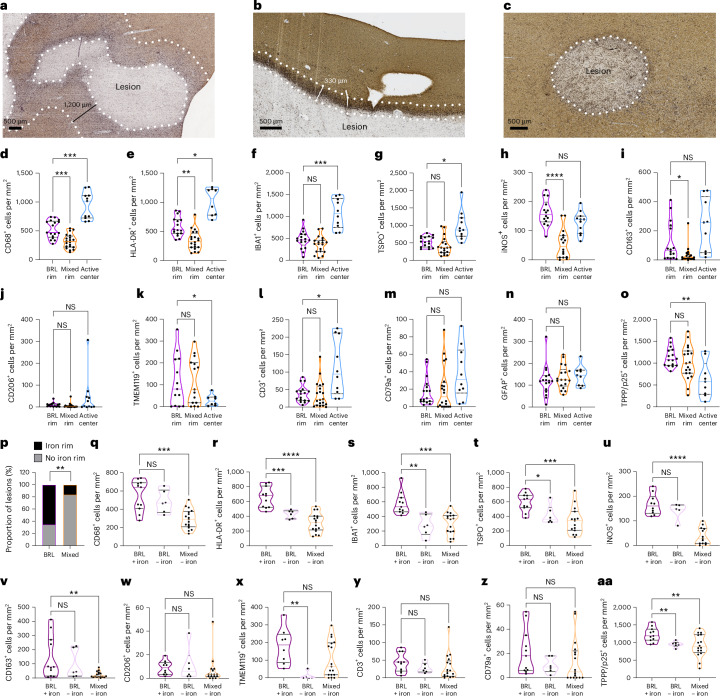
Histopathological characteristics of BRLs in mixed and active lesions. **a**–**c**, BRLs (**a**), classical mixed lesions (**b**) and active lesions (**c**) stained for proteolipid protein (PLP) (brown) and HLA-DR (black). The dashed lines indicate the areas that were analyzed for subsequent quantifications. **d**–**k**, Comparison of densities of CD68^+^ (**d**), HLA-DR^+^ (**e**), IBA1^+^ (**f**), TSPO^+^ (**g**), iNOS^+^ (**h**), CD163^+^ (**i**), CD206^+^ (**j**) and TMEM119^+^ (**k**) cells in the rims of BRLs and of mixed and active lesion centers. **l**–**o**, Densities of CD3^+^ T cells (**l**), CD79a^+^ B cells (**m**), GFAP^+^ astrocytes (**n**) and TPPP/p25^+^ oligodendrocytes (**o**). **p**, BRLs had a higher proportion of iron rims compared to classical mixed lesions**. q**–**aa**, Comparison of myeloid cell features, lymphocyte infiltration and oligodendrocyte numbers in BRLs with and without iron rims and in mixed lesions without iron. CD68^+^ (**q**), HLA-DR^+^ (**r**), IBA1^+^ (**s**), TSPO^+^ (**t**), iNOS^+^ (**u**), CD163^+^ (**v**), CD206^+^ (**w**) and TMEM119^+^ (**x**) cells, and CD3^+^ T cells (**y**), CD79a^+^ B cells (**z**) and TPPP/p25^+^ oligodendrocytes (**aa**). **d**–**o**,**q**,**aa**, In the violin plots, *P* values were determined, depending on normality, using a Brown–Forsythe or a one-way Welch’s ANOVA and Dunnett’s test for multiple comparisons (**d**,**f**,**k**,**o**,**r**–**u**,**aa**) or Kruskal–Wallis test (one-way ANOVA) and Dunn’s multiple comparison test (**e**,**g**–**j**,**l**–**n**,**q**,**v**–**z**). **p**, The *P* value was determined using a Fisher’s exact test. *P ≤ 0.05; **P ≤ 0.01; ***P ≤ 0.001; ****P < 0.0001. Source data

**Extended Data Fig. 1:**
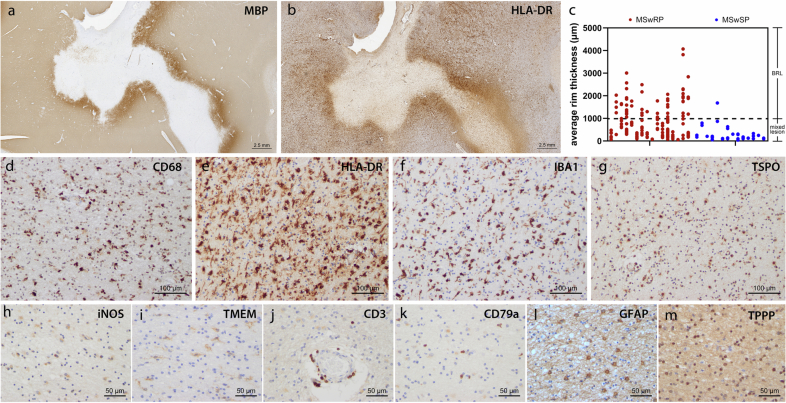
Histological characteristics of broad rim lesions (BRL). Large demyelinated lesion with a sharp border (immunohistochemistry for MBP). **b**, the lesion is surrounded by a broad rim of HLA-DR positive myeloid cells. **c**, quantification of myeloid rim size in a subset of MSwRP and MSwSP. **d** to **g**, Immunohistochemistry for CD68+ (**d**), HLA-DR+ (**e**), IBA1+ (**f**) myeloid cells as well as TSPO+ myeloid cells and astrocytes (**g**). **h** to **m**, Depiction of iNOS+ (**h**) and TMEM119+ cells (**i**), CD3 + T cells (**j**), CD79a positive B cells (**k**), GFAP+ astrocytes (**l**) and TPPP/p25+ oligodendrocytes (**m**). Scale bars in **a** and **b**: 2.5 mm, scale bars in **d** to **g** 100 µm, scale bars in **h** to **m** 50 µm.

**Extended Data Fig. 2:**
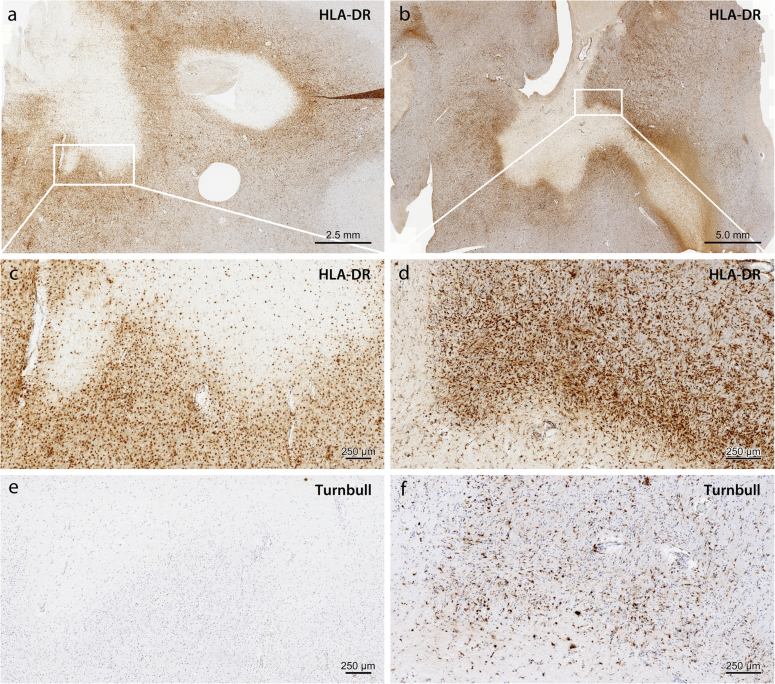
Broad rim lesions (BRL) with and without iron rims. The left panel displays a BRL lesion without an iron rim, whereas the right panel displays a BRL lesion with an iron rim. **a** and **b**, Two broad rim lesions stained for HLA-DR. **c** and **d**, Inserts in **a** and **b** are shown in higher magnification in **c** and **d**. **e** and **f**, Turnbull staining reveals a high number of iron containing myeloid cells at the border of the lesion in **f**, but not in **e**. Scale bars in **a** 2,5 mm, in **b** 5 mm, and in **c** to **f** 250 µm.

**Extended Data Fig. 3:**
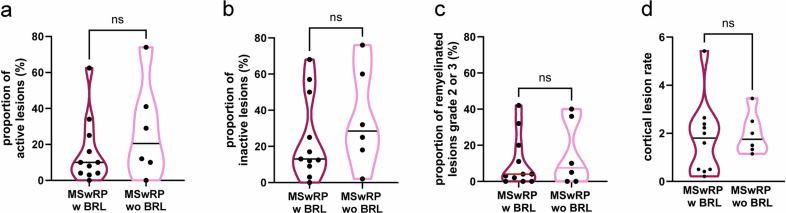
Comparison of the histopathological characteristics of MS patients with rapid disease progression (MSwRP) with and without BRL. **a** to **c**, MSwRP with and without BRL had comparable proportions of active and inactive lesions. **c**, only a small subset of lesions in MSwRP display signs of marked remyelination independent of the presence or absence of BRL; **d**, No difference in the cortical lesion rate between MSwRP with and without BRL; **a** to **d**: violin plots, two-tailed p-values were determined by Mann-Whitney test. Source data

A subset of lesions displayed a remarkably broad rim of macrophages and microglia at least 1-mm wide, which we termed broad rim lesions (BRLs) (Fig. 3a and Extended Data Figs. 1 and 2a,b). The average BRL rim size (quantified in a subset of *n* = 54 lesions) was 1,761 μm and the average mixed-lesion rim size (quantified in a subset of *n* = 158 lesions) was 344 μm (Extended Data Fig. 1c). BRLs were identified in 11 of 17 MSwRP patients, but only 1 of 26 MSwSP patients (Fig. 2w). BRLs accounted for 8–68% (median 27.0%) of total lesions in patients with BRLs (Fig. 2x). Patients in the MSwRP group with or without BRLs showed no significant differences in the proportions of active or inactive lesions, remyelination or cortical lesion rates (Extended Data Fig. 3a–d).

### Perilesional rims of myeloid cells and disease progression

**Extended Data Fig. 4:**
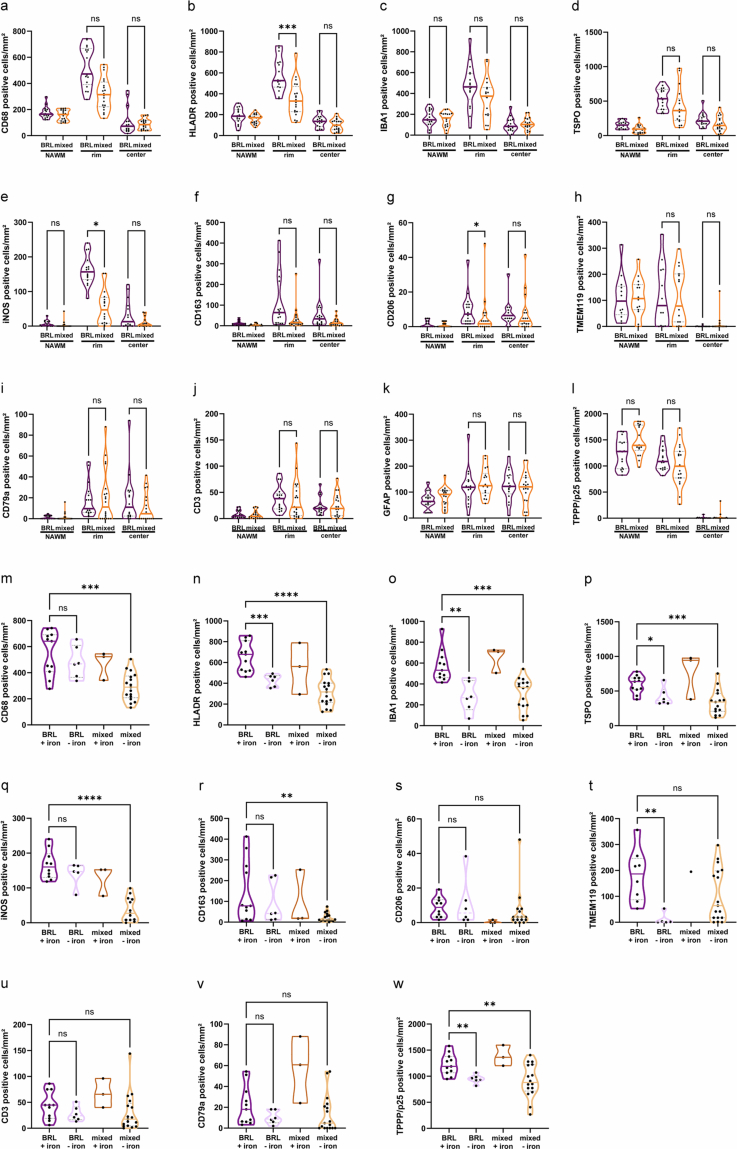
Comparison of BRL and mixed lesions. **a** to **a**, Comparison of densities of CD68^+^ (**a**), HLA-DR^+^ (**b**), IBA1^+^ (**c**), TSPO^+^ (**d**), iNOS^+^ (**e**) 163^+^ (**f**) CD206^+^ (g) and TMEM119^+^ (h) cells in NAWM, rims and lesion centers of BRL and mixed lesions. **i** to **k**, No differences in the densities of CD79a^+^ B cells (i), CD3^+^ T cells (**j**), GFAP^+^ astrocytes (**k**) and TPPP/p25^+^oligodendrocytes (**l**) in the rims of BRL. **m** to **w**, Stratification of BRL and mixed lesions with respect to the presence or absence of iron depositions in the rims of these lesions. Densities of CD68^+^ (**m**), HLADR^+^ (**n**), IBA1^+^ (**o**), TSPO^+^ (**p**), iNOS^+^ (**q**), CD163^+^ (**r**), CD206^+^ (**s**), TMEM119^+^ (**t**), CD3^+^ (**u**), CD79a^+^ (**v**) and TPPP/p25^+^ (**w**) cells a to w: violin plots, p values were determined depending on normality by Kruskal-Wallis (one-way ANOVA) and Dunn’s multiple comparison test (a, c to j, i,r to v) or Brown-Forsythe and Welch ANOVA test (one-way ANOVA) and Dunnett’s correction for multiple comparisons (b,c, k, m to o and w). * p ≤ 0.05, ** p ≤ 0.01, *** p ≤ 0.001, **** p ≤ 0.0001. Source data

We next conducted a detailed characterization. First, we compared the histopathological features of BRLs (*n* = 17) with classical mixed lesions (*n* = 20) and active lesions (*n* = 11) (Fig. 3a–c). We quantified myeloid cells, T cells, B cells, astrocytes and oligodendrocytes in the normal-appearing white matter (NAWM), and rim and lesion centers (Fig. 3 and Extended Data Figs. 1 and 4). Active lesion centers had the highest densities of CD68^+^, HLA-DR^+^ and IBA1^+^ myeloid cells (Fig. 3d–f). BRL rims had higher densities of CD68^+^ and HLA-DR^+^, but not IBA1^+^, myeloid cells compared to mixed-lesion rims (Fig. 3d–f). Translocator protein 18 kDa (TSPO) is expressed in myeloid cells and astrocytes and TSPO positron emission tomography (PET) is used to detect inflammatory activity in the brain^12,13^. TSPO^+^ cells were abundant in all lesion types, highest in active lesions, but similar in BRL and mixed-lesion rims (Fig. 3g). BRL rims had significantly higher densities of iNOS^+^ and CD163^+^ cells than mixed-lesion rims (Fig. 3h,i). Few myeloid cells expressed the anti-inflammatory marker CD206 (Fig. 3j), with no difference between lesion types. TMEM119^+^ microglia were lowest in active lesion centers, with no differences in BRL and mixed rims (Fig. 3k). Active lesions had more CD3^+^ T cells and fewer TPPP/p25^+^ oligodendrocytes compared to BRL and mixed rims, but T cell, B cell, astrocyte and oligodendrocyte counts were comparable between BRL and mixed lesions (Fig. 3l–o). No differences were noted between the lesion centers and NAWM of mixed and BRL lesions (Extended Data Fig. 4a–l).

Iron deposition at lesion borders in imaging is linked to disease progression^14,15^. Using Turnbull staining, we characterized iron status in the rim of BRLs and mixed-lesion rims (Fig. 3 and Extended Data Figs. 2 and 4). Iron rims covering more than 50% of the lesion circumference were present in 11 of 17 BRL and 3 of 19 mixed lesions (Fig. 3p and Extended Data Figs. 2 and 4). Iron^+^ BRL rims had higher densities of CD68^+^, HLA-DR^+^, IBA1^+^, TSPO^+^, iNOS^+^ and CD163^+^ cells compared to iron^−^ mixed lesions (Fig. 3q–v). Iron^+^ BRLs also had higher densities of HLA-DR^+^, IBA1^+^, TSPO^+^ and TMEM19^+^ cells than iron^−^ BRLs (Fig. 3r–t,x). No differences were observed for CD206^+^ myeloid cells, CD3^+^ T cells and CD79a^+^ B cells between lesions (Fig. 3w,y,z). TPPP/p25^+^ oligodendrocytes were higher in iron^+^ BRL rims, suggesting that iron may mark earlier lesion stages with preserved oligodendrocytes (Fig. 3aa). The data for mixed lesions with iron rims (*n* = 3) are shown in Extended Data Fig. 4m–w; statistical analyses were not performed because of low numbers.

**Extended Data Fig. 5:**
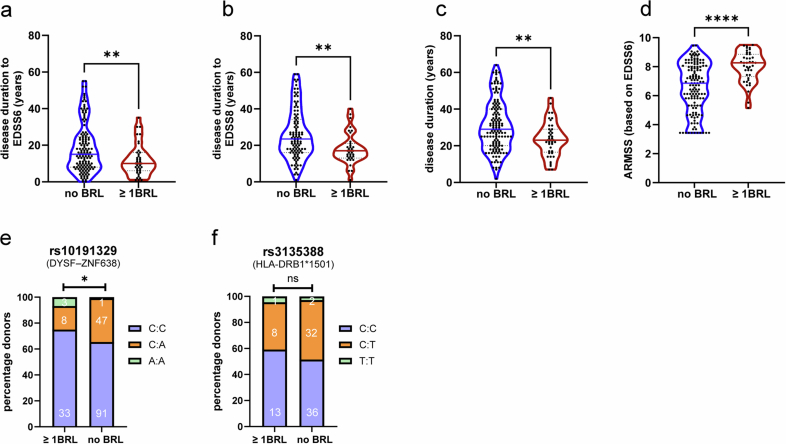
Histological comparison and disease severity and genotype-phenotype correlations within the entire NBB-MS cohort with and without broad rim lesions (BRL). **a** to **c**, Patients with at least one BRL reached EDSS 6 and 8 faster and died after a shorter disease duration compared to patients without BRL, demonstrating that the presence of BRL is associated with a more severe disease course. **d**, Patients with at least 1 BRL have a higher age-adjusted multiple sclerosis severity score (ARMSS). Two-tailed pvalues in a to d were determined by Mann-Whitney test. **e**, Genotype distribution of MS severity associated SNP rs10191329 in MS donors of the NBB with at least 1 BRL or without BRL. The Y-axis displays the relative number of donors carrying the respective genotype and the number in the bar graph represents the absolute number of donors. A significant difference in distribution of genotypes was found between the two groups using Fisher’s exact test (p = 0.015). **f**, Same as e, but for MS susceptibility SNP rs3135388. No significant difference could be detected in the genotype distribution between the two groups (p = 0.60). * p ≤ 0.05, ** p ≤ 0.01, *** p ≤ 0.001, **** p ≤ 0.0001. Source data

In the unselected NBB MS autopsy cohort (*n* = 198), 45 patients (22.7%) had at least one BRL. These patients reached EDSS 6 and 8 faster and had shorter disease durations (Extended Data Fig. 5a–c), supporting the association between BRLs and rapid progression. Patients with BRLs also had significantly higher ARMSS (Extended Data Fig. 5d). Genetic analysis revealed no difference in the susceptibility single-nucleotide polymorphism (SNP) with the largest effect size rs3135388 (HLA-DRB1*1501), but a higher frequency of homozygous carriers of the only known severity SNP rs10191329 (DYSF-ZNF638) in patients with BRLs (Extended Data Fig. 5e,f)^16,17^.

### Transcriptomic signatures

**Fig. 4:**
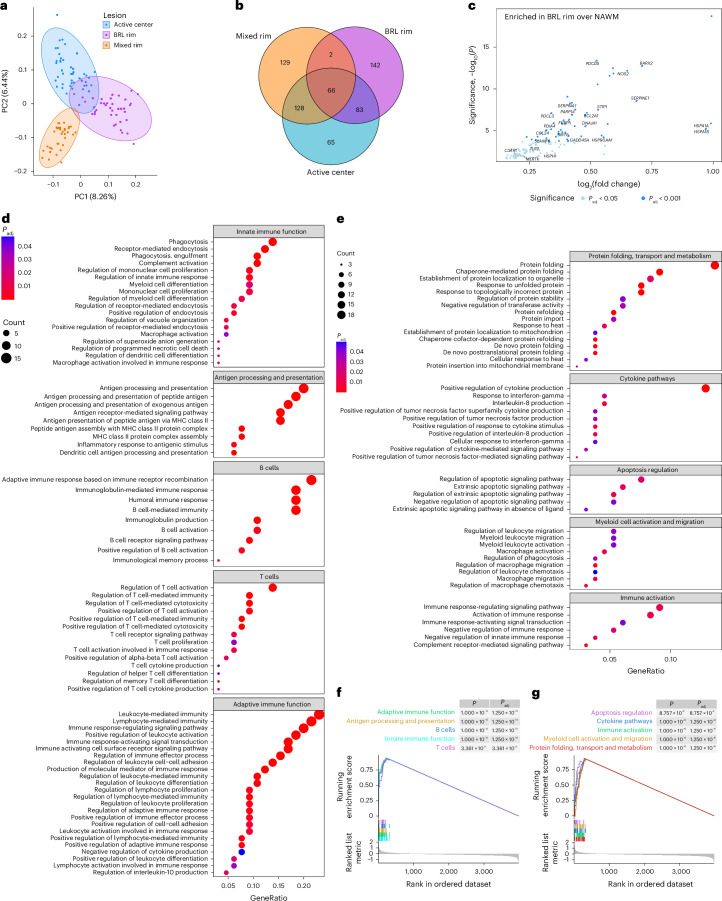
Spatial transcriptomic analysis of CD68^+^ myeloid cells in BRL lesion rims, mixed-lesion rims and active lesion centers compared to NAWM. **a**, PCA of the normalized expression data for the three lesion types (active center, BRL rim, mixed rim). **b**, Venn diagram of DEGs (each lesion type compared to the respective NAWM) of all three lesion types. **c**, Volcano plot of DEGs (BRL rim over NAWM) that are exclusively upregulated in BRLs but not in other lesion types. The blue dots indicate genes with significant differential enrichment between conditions (*P*_adj_ < 0.05). Relevant genes of interest are annotated. All comparisons used moderated two-sided *t*-statistics with Benjamini–Hochberg adjustments for multiple comparisons; non-exclusive, overlapping DEGs were removed. **d**, GSEA based on DEGs (lesional CD68^+^ cells over corresponding NAWM) that are shared between all three lesion types, with GO Biological Processes (BP) gene sets as signatures; clusterProfiler’s implementation of a one-sided Fisher’s exact test was used for the enrichment analysis, with Benjamini–Hochberg correction for multiple comparisons. Only significantly enriched terms of biological interest were selected for dot plot visualization. The full set of significant GO terms is displayed in Supplementary Table 5. The colors indicate *P*_adj_ values; the dot size depicts the geneRatio (the percentage of genes with core enrichment relative to the size of the full gene set). **e**, GSEA based on DEGs (lesional CD68^+^ cells over corresponding NAWM) uniquely overexpressed in BRLs, with GO BP gene sets as signatures; clusterProfiler’s implementation of a one-sided Fisher’s exact test was used for the enrichment analysis, with Benjamini–Hochberg correction for multiple comparisons. Only significantly enriched terms of biological interest were selected for dot plot visualization; the full set of significant GO terms is displayed in our Supplementary Table 5. The colors indicate *P*_adj_ values; the dot size depicts the geneRatio. **f**,**g**, GSEA plots based on the GSEA GO term analysis depicted in **d**,**e**, respectively; the relevant gene sets were grouped according to relevant functions and genes with core enrichment were combined to form the representative signatures. All *P* values were calculated with the clusterProfiler GSEA permutation test and adjusted for multiple hypothesis testing with the Benjamini–Hochberg method. MHC, major histocompatibility complex. Source data

**Extended Data Fig. 6:**
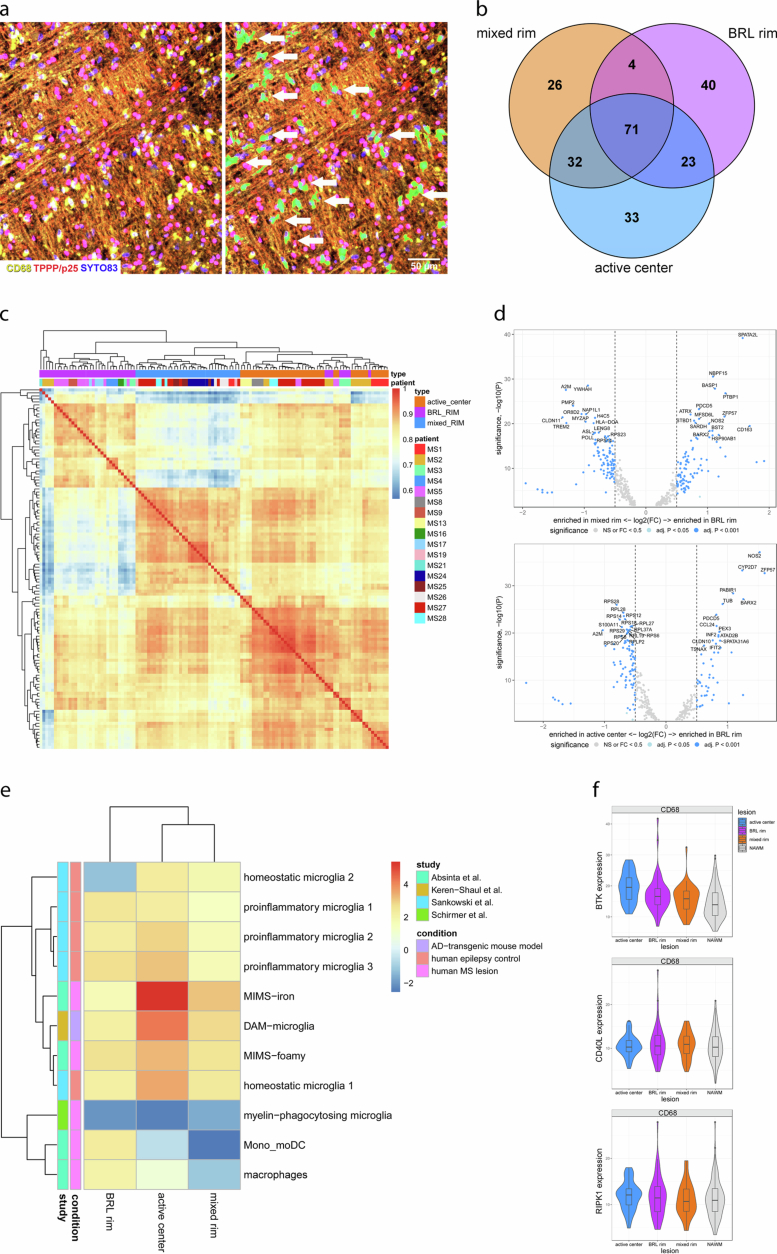
Spatial transcriptomic analysis of CD68 pos myeloid cells in BRL lesion rims, mixed lesion rims and active lesion centers as compared to normal appearing white matter (NAWM). **a**, Example of a BRL lesions stained for CD68 (yellow) and the oligodendroglial marker TPPP/p25 (red) and nuclear staining (SYTO83, blue) in the left panel. In the right panel, demonstration of selection masks (green, arrows) used to identify CD68^+^ myeloid cells for spatial transcriptomic analysis. **b**, VENN diagram of GO terms (each lesion type compared to respective NAWM) of all three lesion types. **c**, Spearman correlation of DEG between all lesion types and NAWM. **d**, Volcano plots of DEGs in BRL CD68^+^ myeloid cells versus mixed lesion CD68^+^ myeloid cells (upper panel) as well as of DEG in BRL CD68^+^ cells versus active lesion CD68^+^ myeloid cells (lower panel), and respective NAWM. Blue dots indicate genes with significant differential enrichment between conditions (adjusted p value < 0.05); relevant genes of interest are annotated. **e**, Heatmap visualization of gene set enrichment analysis (GSEA) of the three lesion types with published transcriptomic signatures, with Gene Ontology (GO) Biological Processes (BP) gene sets as signatures. Only signatures with significance in comparison to at least one lesion type are depicted. **f**, Expression of three genes of interest in all three lesion types and in NAWM, depicted are Bruton tyrosine kinase (*BTK*), *CD40L*, and *RIPK1*. Source data

We analyzed the transcriptomic profiles of CD68^+^ myeloid cells (macrophages and microglia) in BRL rims compared to mixed-lesion rims and active lesion centers using spatial transcriptomics (Fig. 4 and Extended Data Fig. 6a). This analysis included 35 MS lesions from 17 donors, including 13 BRLs, 11 mixed lesions and 11 active lesions, and the corresponding NAWM. Myeloid cell signatures exhibited distinct transcriptional profiles across lesion types, as shown by principal component analysis (PCA) (Fig. 4a). While the lesion types shared the expression of several genes, each also displayed unique signatures (Fig. 4b,c, Extended Data Fig. 6b and Supplementary Table 4). Spearman correlation of differentially expressed genes (DEGs) showed greater similarity between BRLs and active lesions than between BRLs and mixed lesions (Extended Data Fig. 6c). Gene Ontology (GO) term analysis of the myeloid cell core signature shared by all lesion types revealed upregulation of adaptive immune functions, including antigen presentation and T and B cell activation (Fig. 4d,f and Supplementary Table 5). In contrast, BRL-specific myeloid cell gene signatures indicated enhanced protein turnover, proinflammatory cytokine production, apoptosis and myeloid cell migration (Fig. 4e,g and Extended Data Fig. 6d). These findings suggest elevated innate immune activity linked to endoplasmic reticulum (ER) stress and the unfolded protein response (UPR), already implicated in different neuroinflammatory diseases, including MS^18,19^. Volcano plots highlight BRL-enriched genes compared to NAWM, including *iNOS*, heat shock protein family members, UPR-related genes (for example, *STIP1* and *PDCL3*), necroptosis-related genes (for example, *CASP1*, *PDCD5*, *SERPINE1* and *TLR2*) and migratory genes (for example, *CCL24* and *SLAMF8*) (Fig. 4c). Mixed lesions lacked distinct transcriptional signatures despite similar DEG counts relative to NAWM (Fig. 4b and Supplementary Tables 4 and 5). Heatmap visualization of gene set enrichment analysis (GSEA) using published myeloid and microglial signatures showed enrichment of inflammatory microglial subsets (for example, iron-associated microglia inflamed in MS, microglia inflamed in MS (MIMS)-iron and disease-associated microglia) in active lesion centers and, to a lesser extent, mixed-lesion rims. In contrast, BRL myeloid cells were enriched for macrophage and dendritic cell signatures, suggesting similarities with peripheral myeloid cells (Extended Data Fig. 6e and Supplementary Table 6)^20–23^.

Our findings indicate that CD68^+^ myeloid cells in BRL rims exhibit a highly active proinflammatory phenotype linked to ER stress and the UPR, driving strong innate inflammatory and destructive activity.

### BRL detection using TSPO PET and association with progression

**Fig. 5:**
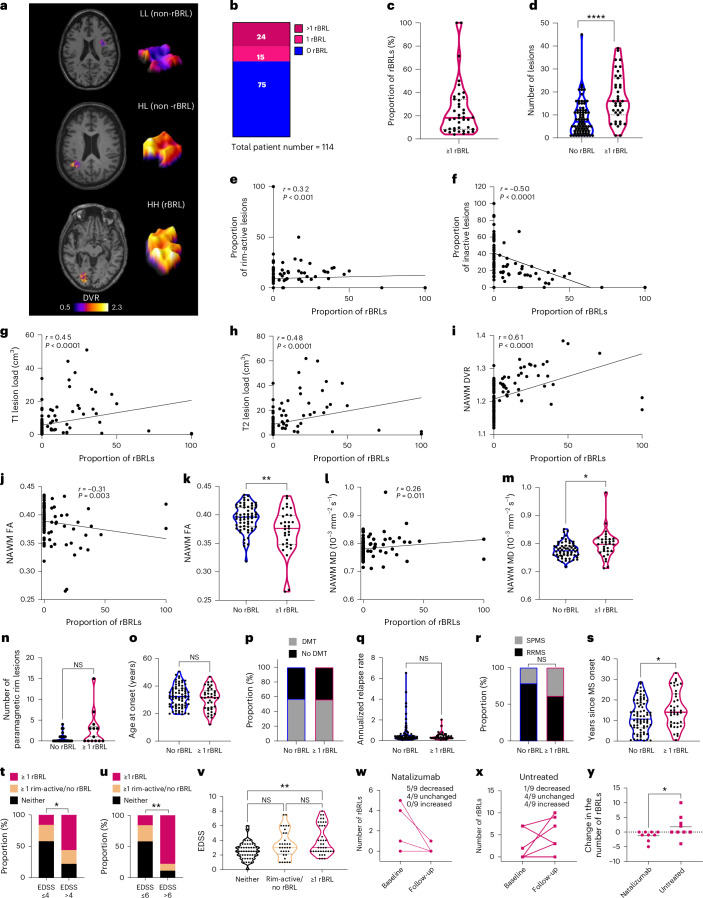
Detection of rBRLs using TSPO PET. **a**, Detection of rBRLs versus non-BRLs using TSPO PET. Examples of rBRL, non-rBRL rim-active and inactive lesions are shown (labeled as HH, HL and LL, respectively). The color bar shows the dynamic range of DVR in the images. **b**, Patients with no, one or more than one rBRL. **c**, Violin plot of the proportion of rBRLs in patients with at least one rBRL. **d**, Total lesion numbers were higher in patients with at least one rBRL (*P* = 1.1 × 10^−6^; Mann–Whitney *U*-test). **e**,**f**, Proportion of rBRLs correlated with the proportion of TSPO PET rim-active lesions (**e**) (*P* = 0.0005) and inversely with the proportion of TSPO PET inactive lesions (**f**) (*P* = 1.1 × 10^−8^) (Spearman correlation). **g**–**i**, Proportions of rBRL correlated with T1 (**g**) (*P* = 4.5 × 10^−7^) and T2 (**h**) lesion loads (*P* = 4.9 × 10^−8^) in MRI and with NAWM DVR (**i**) (*P* = 4.5 × 10^−7^) in TSPO PET (Spearman correlation). **j**–**m**, FA in NAWM (**j**,**k**) correlated negatively and MD in NAWM (**l**) correlated positively with rBRL proportions (Spearman correlation); rBRL patients had lower FA (**k**) *P* = 0.006) and higher MD values (**m**) (*P* = 0.039) in NAWM (Welch’s *t*-test). **n**, Patients (*n* = 41) with at least one rBRL had slightly higher numbers of QSM^+^ rim lesions (*P* = 0.087; Mann–Whitney *U*-test). **o**, Violin plot of age at disease onset in patients with and without rBRLs. An unpaired *t*-test was used. **p**,**q**, Proportions of patients treated with DMTs (Fisher’s exact test) (**p**) and annual relapse rate (**q**) in patients with and without rBRLs (Mann–Whitney *U*-test). **r**, Proportion of SPMS in patients with and without rBRL (*P* = 0.075; Fisher’s exact test). **s**, Disease duration in patients with and without rBRL (*P* = 0.044; Mann–Whitney *U*-test). **t**,**u**, EDSS milestones (EDSS 4 or 6 within 12 years) in patients with rBRL, non-rBRL rim-active lesion or neither (**t**, *P* = 0.028; **u**, *P* = 0.006). A Fisher’s exact test was used. **v**, Higher EDSS in patients with rBRL (*P* = 0.005; Dunn’s test) but not non-rBRL rim-active lesions compared to those without these lesion types (Kruskal–Wallis test, *P* = 0.006). **w**,**x**, Changes in rBRL numbers in natalizumab-treated (**w**) (*n* = 9) and untreated (**x**) MS patients (*n* = 9). **y**, Natalizumab and untreated cohorts in **w**,**x** had a significant difference in the change in rBRL numbers (*P* = 0.021; Mann–Whitney *U*-test). All tests used in this figure are two-tailed. *P ≤ 0.05; **P ≤ 0.01; ****P < 0.0001. Source data

**Extended Data Fig. 7:**
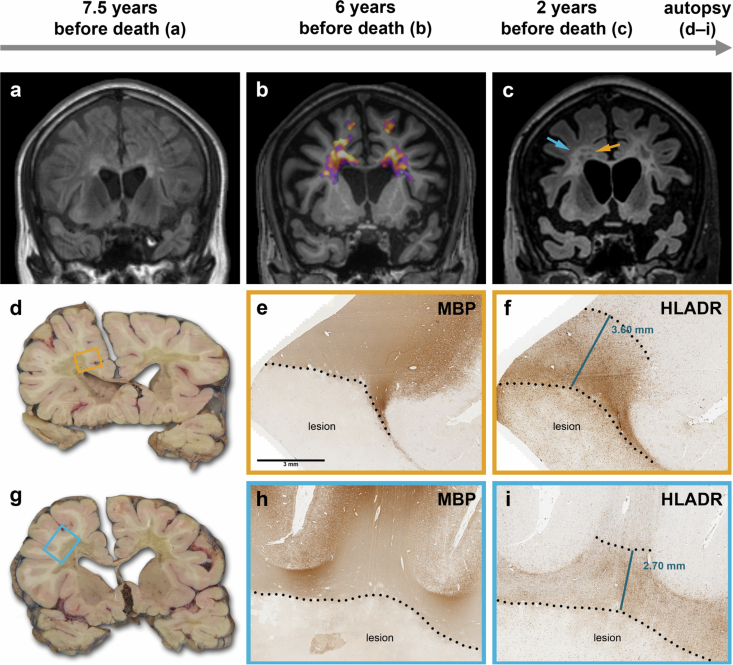
Correlation between imaging findings and histopathology. An autopsy case: a male subject with progressive MS, aged 44.5 years with a disease duration of 15 years at death. **a**, FLAIR image 7.5 years before death showing large lesions. **b**, T1 image 6 years before death overlaid with PET showing high PK11195 signal at the edge of the lesions. **c**, FLAIR image 2 years before death showing further enlargement of the lesions with prominent atrophy. The arrows and their colors refer to **d** and **g. d** and **g**, Gross anatomy. The boxed areas were analyzed by histology. **e** and **f**, The lesion area indicated by the orange rectangle in **d** stained for the myelin marker MBP (**e**) and the myeloid cell marker HLA-DR (**f**). **h** and **I**, The lesion area indicated by the blue rectangle in **g** stained for MBP (**h**) and HLA-DR (**i**). Dashed lines in **e,f**, and **h,i** indicate the lesion border or the rim border, respectively. Scale bar for **e,f,h,i**: 3 mm.

To explore whether BRL could serve as an in vivo biomarker for MS progression, we analyzed a TSPO PET MS cohort (*n* = 114; 32 (28%) males, 82 (72%) females; 31 (27%) with secondary progressive multiple sclerosis (SPMS) and 83 (73%) RRMS patients; Fig. 1). Lesions with a broad perilesional rim of macrophages/microglia were classified as radiological BRLs (rBRLs) based on distribution volume ratio (DVR) patterns in the concentric rims 2-mm wide up to 4 mm around the lesion (this wider width was chosen to account for the known more diffuse PET signal compared to histology). Lesions exhibiting a HIGH-HIGH pattern within each concentric 2-mm segment were classified as rBRLs (Fig. 5a). Among 1,326 white matter (WM) lesions greater than 50 mm^3^, 137 (10.3%) were rBRLs. rBRLs were seen in 39 patients (34.2%); 15 patients had one rBRL and 24 more than one (Fig. 5b). Postmortem analysis confirmed the presence of histological BRLs in one patient (Extended Data Fig. 7).

The proportion of rBRLs of all lesions ranged between 4% and 100% (Fig. 5c). Patients with at least one rBRL had higher lesion counts; higher rBRL proportion correlated with higher TSPO PET rim-active and lower TSPO PET inactive lesion proportions (Fig. 5d–f). Imaging findings are summarized in Supplementary Table 7.

**Extended Data Fig. 8:**
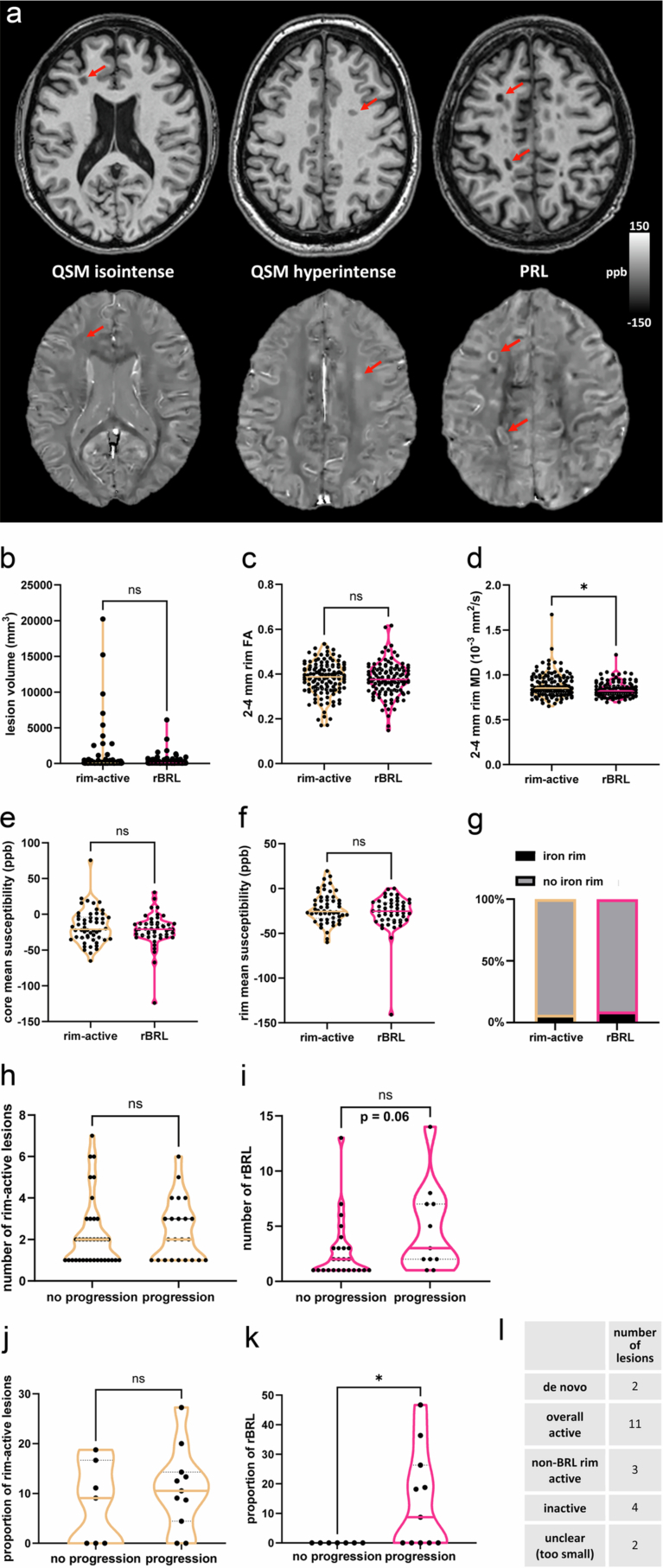
Imaging findings. **a** Examples of T1 and QSM images. Arrows indicate T1 lesions (upper row) and the corresponding sites after QSM analyses (lower row). **b-f**, There was no difference in lesion volumes, 2–4 mm T1 lesion rim FA values, or core and rim susceptibility values between the individual rBRL lesions and non-rBRL rim-active lesions, but rBRL had significantly lower 2–4 mm T1 lesion rim MD values (Mann-Whitney U-test). **g**, There was no difference in the presence of iron rims found in individual rBRL and non-rBRL rim-active lesions (Fisher’s exact test). **h**, Patients with non-rBRL rim-active lesions and progression data from PET imaging onwards available (n = 72): no difference was found in the number of non-rBRL rim-active lesions (Mann-Whitney U-test). **i**, Patients with rBRL and progression data from PET imaging onwards available (n = 36): there was a trend towards the progressed patients having more rBRL (p = 0.066; Mann-Whitney U-test). Mean follow-up time from PET imaging to follow-up EDSS measurement was 5.5 years (SD 2.3). **j**-**k**, Proportion of non-rBRL rim-active lesions (**j**) and rBRL (**k**) in an untreated MS cohort (n = 18) followed up clinically for an average of 6.5 (SD 2.4) years from PET imaging (n = 11 with progression, n = 7 remaining stable). **l**, In reference to Fig. 5x–y, this table shows the baseline lesion phenotype status of the rBRLs detected at follow-up PET imaging at one year time point for the untreated cohort (n = 9). Source data

A strong correlation between the proportion of rBRLs and NAWM PET [^11^C]PK11195 DVR, T1 and T2 lesion volume was observed, indicating that rBRLs are associated with diffuse microglial activation and an increased magnetic resonance imaging (MRI) lesion load (Fig. 5g–i). As a sign of diffuse WM damage, an increase in mean diffusivity and decrease in fractional anisotropy in diffusion tensor imaging (DTI) MRI was found (Fig. 5j–m). From 41 patients (13 with and 28 without rBRLs), data on paramagnetic rim lesions were available. No significant difference in paramagnetic rim lesions was observed between rBRL and non-rBRL patients (*P* = 0.087) (Fig. 5n and Extended Data Fig. 8a). Clinically, rBRL patients showed longer disease duration, but no differences in age at onset, disease-modifying therapy (DMT) use, annualized relapse rate or SPMS proportion (Fig. 5o–s).

At a lesional level, rBRL and non-rBRL rim-active lesions showed no differences in lesion volume, lesional DTI measures or iron content (Extended Data Fig. 8b–g). Patients who progressed from PET imaging onwards showed a trend toward more rBRLs (*P* = 0.066; Extended Data Fig. 8h,i). Furthermore, in a subset of untreated patients, there was a significant association between the presence of rBRL but not non-rBRL rim-active lesions with disease progression from PET imaging onwards (Extended Data Fig. 8j,k). To further evaluate the potential prognostic value of rBRLs, we differentiated between individuals with at least one rBRL, at least one non-BRL rim-active lesion or those lacking both types. Notably, patients reaching EDSS milestones (EDSS > 4, EDSS > 6) within 12 years from disease onset were more likely to have rBRLs compared to non-BRL rim-active lesions or neither (EDSS > 4: rBRL 56%, rim-active 22%, neither 22%; EDSS > 6: rBRL 78%, rim-active 11%, neither 11%; Fig. 5t–v).

Finally, in a longitudinal cohort (*n* = 9) treated with natalizumab, five patients showed reduced numbers of rBRLs after 1 year, while in untreated controls (*n* = 9) four patients showed increased numbers (Fig. 5w–y and Extended Data Fig. 8l). Taken together, our data suggest that in vivo detection of BRLs is feasible using TSPO PET imaging. The presence of rBRLs correlates with several known imaging features associated with disease severity and progression, supporting TSPO PET imaging as a tool for in vivo monitoring of the pathology associated with disease progression.

## Discussion

We identified MS lesions with a broad rim of myeloid cells as a hallmark of rapid disease progression in a subset of patients. These myeloid cells exhibited a distinct inflammatory phenotype at the histopathological and transcriptional levels. Furthermore, our data suggest that TSPO PET imaging enables in vivo detection of BRLs; rRBLs are a potential biomarker for inflammation-driven progression and aid in targeting myeloid cell-driven inflammation in the CNS.

Analysis of a well-characterized MS autopsy cohort with extensive clinical data allowed for donor stratification according to progression dynamics. Consistent with earlier findings, rapidly progressing patients showed more active and mixed lesions, less remyelination and a higher prevalence of brain stem and spinal cord lesions, confirming previous associations with severe disability accumulation^24–28^. A recent study identified the first genetic association with disease severity in MS (SNP rs10191329)^17^. Notably, this SNP has not only been found at an increased frequency in NBB donors exhibiting BRLs but has also been linked to an elevated burden of brain stem and cortical lesions, suggesting a genetic influence on lesion development in MS with contribution to disease progression.

Consistent stratification based on progression dynamics facilitated the discovery of an enrichment of MS lesions characterized by a particularly broad myeloid cell rim observed almost exclusively in patients with rapid progression. Compared to classical mixed lesions, BRLs displayed higher densities of CD68^+^, HLA-DR^+^, CD163^+^ and iNOS^+^ cells, alongside a higher proportion of iron rims. Transcriptomic analysis of myeloid cells across all lesion types revealed a signature characterized by enrichment in adaptive immune responses, such as antigen processing and presentation, and support of and response to T and B cells. This supports the idea that MS lesion-associated myeloid cells are intricately involved in the reciprocal interaction with T and B cells and this may contribute to lesion propagation or prevention of resolution of inflammation. This strongly supports the relevance of drug interventions targeting myeloid cell-lymphocyte interactions at the lesional level. One example could be the therapeutic inhibition of CD40L signaling, such as with the antibody frexalimab, although its CNS penetrance is uncertain. Nonetheless, frexalimab has shown promising first results in a phase II trial for MS and is undergoing phase III trials for relapsing and non-relapsing progressive forms of MS^29^. However, myeloid cells from BRLs displayed unique signatures pointing toward enhanced protein synthesis and turnover, leading to ER stress, upregulated UPR, proinflammatory cytokines, including interleukin-8, and receptor-interacting serine/threonine-protein kinase 1 (RIPK1)-associated pathways, such as apoptosis and necroptosis. These pathways are not only implicated in MS progression but also in neurodegenerative diseases like Parkinson’s and Alzheimer’s and warrant further investigation as potential therapeutic targets^18,30–34^. Of note, Bruton’s tyrosine kinase (BTK) inhibitors have been shown to affect UPR signaling, Toll-like receptor 2 and RIPK1-related pathways while mitigating interleukin-8 production in different conditions, but this has not been addressed in MS so far^35–37^. Recent results on the BTK inhibitor tolebrutinib demonstrated efficacy on clinical measures of disability progression in relapsing MS and non-relapsing secondary progressive MS^38^. Similarly, a RIPK1 inhibitor was tested in a phase II trial of MS, although the trial was terminated because it failed to meet its primary endpoint, that is, neurofilament light chain levels^39^. We identified the genes of the mentioned molecular targets CD40L, BTK and RIPK1 using spatial transcriptomics in all MS lesion types investigated (Extended Data Fig. 6f).

Earlier studies aimed to identify the molecular characteristics of several lesion types and compared them to NAWM and control tissue using different techniques^20,40–45^. These studies identified MS-specific astrocytic, microglial and oligodendroglial subpopulations^20,43,45^ and distinct cellular signatures associated with mixed and inactive lesions^20^. A recent study identified lesion-specific microglial signatures and confirmed the presence of transcriptomic signatures of MIMS-iron and MIMS-foamy in mixed lesions, but did not investigate BRLs or stratify donors according to clinical trajectories^46^. Heterogeneity and limited sample size, combined with lack of spatial resolution and stratification according to clinical parameters, have hindered detection of signatures linked to disease evolution at the patient level.

Strong TSPO expression in BRL-associated macrophages and microglia prompted the use of TSPO PET imaging for the evaluation of an in vivo correlate of BRL. Indeed, TSPO PET imaging has identified innate immune cell activation in MS lesions and correlated TSPO signals in MS lesions with disease progression^47^. Imaging revealed a significant correlation between perilesional macrophage and microglia rim width and disease progression speed, supporting the concept of myeloid cell rim width as a biomarker of progression. Notably, treatment strategies preventing immune cell migration into the CNS, such as natalizumab, can significantly influence the persistence of rBRLs, as several rBRLs were absent after 1 year of treatment, thus highlighting the peripheral immune system’s relevance in MS lesions linked to disease progression.

Over half of the BRLs examined contained iron rims, which are associated with higher myeloid cell densities and increased innate inflammation compared to BRLs without iron rims, suggesting a link between iron deposition and the degree of innate inflammation. In contrast, iron deposition was rare in classical mixed lesions. Iron-containing phase-rim MRI lesions have been identified as significant predictors of disability progression^48,49^; it is plausible that these overlap with iron-containing BRLs, representing a particularly detrimental myeloid cell-driven inflammatory lesion type linked to MS progression. However, at the lesion level, only a minority of rBRL and non-rBRL rim-active lesions were iron^+^ based on magnetic susceptibility imaging. Two factors may account for this observation. First, MRI may be less sensitive in detecting iron deposition compared to histological methods. Second, there may be limited overlap between iron-depositing lesions and those with a broad myeloid cell rim, a phenomenon also noted with the poor overlap of phase-rim and slowly expanding lesions, both associated with disease progression^50,51^. It is conceivable that BRLs constitute a subset of slowly expanding lesions, as suggested by findings from our autopsy case. Further research is necessary to better correlate histological iron-containing lesions with MRI-defined phase-rim lesions, slowly expanding lesions and TSPO PET imaging to deepen our understanding of their roles in disease progression and contributions to disease heterogeneity.

We identified BRLs in 11 of 17 patients with rapid progression, indicating that other mechanisms might drive progression in a sizeable portion of cases. The concept of distinct pathologies underlying MS subtypes as a driver of disease heterogeneity has been proposed before, including a recent MRI study using unsupervised machine learning to identify different MS subtypes and another study using factor analysis-determined dimensions in a postmortem pathology cohort^52,53^. We have also contributed to this concept by characterizing distinct peripheral blood immune signatures in early relapsing MS, which are associated with specific disease trajectories and potential treatment responses^54^.

In the future, it will be critical to prospectively determine these distinct pathomechanisms in individual patients using advanced imaging technologies in combination with other biomarkers. This approach will enable optimal patient stratification in clinical trials investigating new anti-inflammatory or neuroprotective treatments, enriching for potential responders and thereby reducing the risk of negative outcomes for promising therapeutic approaches.

While we focused on extreme clinical phenotypes of disease progression in our well-characterized cohort, we acknowledge that the granularity of the clinical data cannot fully exclude an additional focal inflammatory component contributing to disability accumulation in our rapidly progressing group. Nevertheless, clinical summaries recorded deterioration in functioning in the absence of recorded attacks. Accordingly, no difference in relapsing onset was noted between the two groups.

We also acknowledge the absence of longitudinal data in our autopsy cohort, which limits our ability to determine the origin and fate of BRLs. One possible explanation is that BRLs represent a post-acute stage of lesion evolution, arising from WM lesions in younger individuals with a genetic or other predisposition to an exaggerated innate immune response. These lesions may then follow diverse trajectories, including iron depletion, as suggested by the observed variability in BRL iron content. Additionally, it is possible that BRLs may either originate from or transition into mixed lesions. In our imaging cohort, longitudinal analysis of untreated patients suggests that rBRLs can occur de novo, and arise from previously existing lesions, in particular overall active lesions (Extended Data Fig. 8). However, while we were able to correlate PET and histology findings in a single patient, additional studies are warranted to further confirm that rBRLs accurately represent histological BRLs.

In summary, we have demonstrated that in a subset of patients with MS, rapid disease progression is associated with pronounced activation of myeloid cells located in a broad perilesional rim. These findings suggest that such patients could benefit from pharmacological treatments targeting the innate immune response in myeloid cells, a therapeutic target that is not adequately addressed by current treatment options. Our findings further highlight the importance of developing advanced imaging tools and biomarkers to identify the dominant pathological mechanisms driving disease progression in individual patients with MS. This, we predict, will be critical for identifying new therapeutic targets and for developing new personalized treatment strategies tackling disease progression in MS.

## Methods

### Ethics oversight

We used archival samples. The research on anonymization was in accordance with local ethical standards and regulations by the Ethics Committee of the Vrije Universiteit Amsterdam Medical Center, the Ethics Committee of the Hospital District of Southwest Finland and the Ethics Committee of the University Münster.

### PET data

All data from study participants included in the PET analyses were acquired according to study protocols previously approved by the Ethics Committee of the Hospital District of Southwest Finland. All study participants provided written informed consent according to the principles of the Declaration of Helsinki (2008).

### Identification of patients with MS with a very slow or very rapid disease progression

The narratives of the NBB summaries of medical dossiers (*n* = 186) were screened by two experienced MS neurologists (L.K. and J.S.). Documented MS severity was estimated based on patient narratives, along with the dates and ages of registration of functional equivalents of the EDSS, such as appearance of walking difficulties (EDSS 3), use of a unilateral walking aid (EDSS 6), restriction to a wheelchair (EDSS 8–9) and death. As a guideline for the definition of severe MS, cases had a progressive course, resulting in death before 50 years of age or an EDSS of 8–9 or death within 15 years of disease onset. Mild cases had a progressive course resulting in death when older than 70 years or an EDSS of 8–9 or death not earlier than 40 years after disease onset. The sex of the participants was determined using the autopsy record.

### Histopathological characterization of MSwSP and MSwRP

Tissue blocks from the MSwSP (*n* = 26) or MSwRP (*n* = 18) were included. Lesion type and extent of remyelination was determined on tissue blocks from the brain, cerebellum and brain stem based on sections stained using immunohistochemistry (IHC) for PLP and HLA-DR-DQ-DP (subsequently termed HLA-DR) in the MSwRP (*n* = 18) and MSwSP (*n* = 21) groups, with on average 18 ± 12 lesions per donor (Fig. 2j)^10^. Active lesions were hypercellular and characterized by a diffuse infiltration of the complete lesion area with numerous HLA-DR^+^ myeloid cells without myelin basic protein (MBP)^+^ myelin degradation products in their cytoplasm (active and post-demyelinating lesions). Mixed active and inactive lesions (also called chronic active lesions) were characterized by a hypocellular lesion center and a rim of activated myeloid cells at the border of the lesion. Inactive lesions were hypocellular with few myeloid cells present in the lesion center^10^. NAWM was defined as non-demyelinated WM with maximal distance to the lesion on the individual tissue block. The extent of remyelination was determined in sections stained for PLP or Luxol fast blue by a semiquantitative score: 0 = no or minimal remyelination at the lesion border of less than 10% of the whole lesion area; 1 = remyelination in more than 10%, but less than 50% of the whole lesion area, 2 = more than 50% of the lesion remyelinated; and 3 = completely remyelinated lesion^11^. To determine the brain stem lesion load, the whole and lesion areas were determined in sections stained for PLP or Luxol fast blue-periodic acid–Schiff using ImageJ. If more than one brain stem tissue block per patient was available, the average brain stem lesion area per patient was determined. Spinal cord demyelination was estimated in PLP-stained sections using a semiquantitative score: 0 = no demyelination; 1 = demyelination of up to 20%; 2 = demyelination of 21–40%; 3 = demyelination of 41–60%; 4 = demyelination of 61–80%; and 5 = demyelination of 81–100% of the cervical, thoracic, lumbar and sacral spinal cord. Tissue sections from all spinal cord levels were not available for all patients. If more than one tissue block per spinal cord level per patient was available, the average demyelination score was determined. Cortical gray matter lesions were identified in PLP-stained sections. To account for a variable number of supratentorial blocks sampled between individuals, cortical lesions were considered as a rate by adding the number of tissue blocks with visible cortex as an offset^17^. Genotyping for rs10191329 was performed using the Kompetitive Allele Specific PCR genotyping platform (LGC Genomics); the tagging SNP rs3135388 for HLA-DRB1*1501 was genotyped using the Infinium Global Screening Array (Illumina, v.3) by the Human Genomics Facility at the Erasmus University Medical Center. The NBB donor program and all procedures of the NBB have been approved by the Ethics Committee of the Vrije Universiteit Amsterdam Medical Center (2009/148).

### Histopathological comparison of BRLs, classical mixed lesions and active lesions

We defined BRLs as mixed active and inactive lesions with a myeloid rim with a rim size of at least 1 mm. For detailed histopathological characterization, we selected 41 formalin-fixed, paraffin-embedded tissue blocks from 29 patients containing in 17 BRLs, 20 mixed lesions and 11 active lesions (Supplementary Table 1). Tissues were cut into 4-µm-thick sections. IHC was performed using the DetectionLine System (cat. no. PD000RP, DCS) and an automated immunostainer (Autostainer Link 48, Dako). IHC was performed using a biotin-streptavidin technique. Primary antibodies were applied as listed in Supplementary Table 2. IHC was completed using biotinylated secondary anti-mouse, rat or rabbit antibodies followed by incubation with streptavidin–peroxidase complex; the reaction product was developed with 3,3′-diaminobenzidine (DAB).

For the detection of total (ferric and ferrous) non-heme iron, DAB-enhanced Turnbull staining was used^55^.

For quantification, sections were analyzed at ×40 or ×100 magnification using a morphometric grid. At least ten visual fields per ROI (lesion center, lesion rim, NAWM) were analyzed. For each ROI, the average counts per square millimeter were calculated and compared using statistical analysis. For the rim size measurements, archive sections of MSwSP and MSwRP donors from the NBB, stained using IHC for PLP, HLA-DR or both, containing subcortical classical mixed or BRLs were scanned using the Axioscope microscope Z1 (ZEISS) with a ×20 objective and 0.8 numerical aperture plan-apochromat and bright field camera (HV-F203SCL, Hitachi). The lesion rims were outlined using QuPath v.0.5.1 based on the outer edge HLA-DR^+^ cells that were distinguishable from perilesional NAWM and the PLP edge. Borders that could not be assigned unequivocally to one hypocellular lesion core, were adjacent to cortical gray matter or without perilesional NAWM were excluded from the measurement. The Interedge Distance 2.0 macro for ImageJ was used to determine the shortest distance between the two edges every 100 µm along the outer edge.

### Transcriptomic analyses

For the transcriptomic analyses, 30 tissue blocks from 17 patients were included containing 13 BRLs, 11 mixed and 11 active lesions and NAWM (*n* = 29) (Supplementary Table 1). Work was performed under RNase-free conditions; 5-µm-thick tissue sections were placed on SuperFrost Plus slides (cat. no. 631-0108, VWR). After deparaffinization, antigen retrieval was performed in a steam cooker using target retrieval solution (10× EDTA pH 9.0, cat. no. 00-4956-58, Invitrogen). Samples were treated with 0.1 µg ml^−1^ Proteinase K (cat. no. 25530-049, Invitrogen) for 15 min at 37 °C and post-fixated with 10% neutral buffered formalin (NBF) (cat. no. F5554, VWR) followed by neutral buffered formalin stop buffer twice (0.1 M glycine, 0.1 M Tris) and phosphate/stop buffer twice (0.1 M glycine, 0.1 M Tris) and PBS, 5 min each. Human Whole Transcriptome Atlas probe reagent (cat. no. 121401102, NanoString) was diluted in buffer R (cat. no. 121300313, NanoString). Slides were covered with HybriSlip covers (Grace Bio-Labs) and incubated for 15–20 h at 37 °C in a ThermoBrite Hybridization oven, humidified with diethylpyrocarbonate-treated water. Coverslips were detached by washing two times with SSC (cat. no. S6639, Sigma-Aldrich), followed by four times with SSC and formamide (cat. no. A2156.1000, VWR) at 37 °C for 25 min. Slides were washed two times in SSC and blocked with 200 µl buffer W (cat. no. 121300313, NanoString) for 30 min followed by incubation with anti-CD68 (1:100 dilution, cat. no. sc-20060AF594, Santa Cruz Biotechnology) and a nuclear stain (SYTO83, 1:10,000 dilution, cat. no. S11364, Thermo Fisher Scientific) diluted in buffer W. Sections were washed two times with SSC buffer for 5 min each. For GeoMx ROI selection, segmentation and collection slides were covered with buffer S (cat. no. 100474, NanoString) and loaded into the GeoMx DSP. For each sample, four ROIs (660 × 785 µm) per region (rims of BRLs and mixed lesions, active lesion center, NAWM) were selected and segmented into areas of interest using the morphology marker CD68. After ultraviolet light exposure, cleaved tags were aspirated, collected and stored at −20 °C or directly dried overnight using a semipermeable AeraSeal film (cat. no. 903172, NanoString). While this approach allows for a strong enrichment of genes in CD68 cells, we cannot fully exclude that some genes from other cell types may have also been analyzed.

### Library preparation and sequencing

PCR amplification was performed according to the manufacturer’s instructions with 4 µl resuspended oligonucleotide tags, 4 µl of unique primers and 2 µl of the GeoMx Seq Code PCR Master Mix (cat. no. 121400205, NanoString). Samples were pooled per plate and purified using two rounds of AMPure XP bead cleanup (cat. no. A63880, Beckman Coulter). Afterwards, sequencing was performed using the Illumina platform. For the next-generation sequencing (NGS) readout, the photocleaved oligonucleotides of the GeoMx collection plate were pooled and used for PCR amplification to add Illumina indexing sequences and create an NGS library. In addition, one non-template control per plate was processed. After library preparation, quality control and quantification was performed using a High Sensitivity DNA 1000 kit on a 4200 TapeStation instrument (Agilent Technologies). Equimolar pooled libraries were used for the sequencing reaction in paired-end mode running 27 cycles of standard sequencing by synthesis chemistry on a NextSeq 2000 instrument (Illumina).

All raw FASTQ files were processed with the NanoString GeoMx NGS Pipeline v.2.3.3.10 on a Linux-based server; counts per gene were exported as NanoString DCC files per sample. The count files were imported into R v.4.2.1 using the Bioconductor package GeomxTools v.3.0.1 (R package v.3.0.1) and the Probe Kit Configuration file Hs_R_NGS_WTA v.1.0. For the initial quality control, the minimum negative control count threshold was set to 1, while all other parameters were kept at their default values. Only samples with a gene detection rate greater than 0.03 were selected for further analysis. For the integrated analysis of the two NanoString runs, the Bioconductor package standR (v.1.6.0)^56^ was used to assess the performance of different batch effect removal strategies. Batch effects were corrected with the standR geomxBatchCorrection function, using the RUV4 algorithm with *k* = 5 and negative control genes based on the GeoMx data slides. Normalization of data was performed using the standR geomxNorm normalization function with the trimmed mean of M-values method; dimensionality reduction was performed using PCA. The Bioconductor pipeline limma v.3.52.4 was used to identify the DEGs between the lesion rims and NAWM samples (false discovery rate < 0.05), and between lesion types^57^. DEGs were visualized with a ggplot2-based volcano plot (v.3.4.4); additional genes of interest with a false discovery rate lower than 0.001 were marked in the volcano plot according to their level of significance.

GSEAs were calculated with the Bioconductor package clusterProfiler v.4.7.112 and the GO BP database. Only gene sets with a minimum size of ten, a maximum size of 500 genes and a *P*_adj_ < 0.05 were considered further. GO terms with significant enrichment were depicted as dot plots to show normalized enrichment scores, *P*_adj_ values and gene ratios indicating the percentage of genes with core enrichment for the respective gene sets.

Based on the DEG results between lesions and the NAWM, common lesion-related genes were defined as genes that were significantly upregulated for each of the three lesion types compared to the NAWM. Genes with a significant enrichment for only one lesion type were defined as lesion-specific genes for the respective lesion type. Any overlap of DEGs between lesion types was visualized as a Venn diagram with the R package VennDiagram v.1.7.3 (ref. ^58^). Furthermore, violin plots for the chosen genes of interest were created with ggplot2. A PCA based on the normalized expression data for each sample was visualized with the same package. Based on the combined lists of DEGs between lesions, a filtered expression matrix was created and pairwise Spearman correlation coefficients were calculated with the basic R cor function. The correlation matrix was visualized as a heatmap with pheatmap v.1.0.12 (https://github.com/raivokolde/pheatmap) using unsupervised clustering for rows and columns based on the pheatmap default Euclidean distance and the complete clustering method. GO and GSEA analyses were performed for the full dataset, and further GSEA enrichments were calculated for myeloid cell-related signatures from refs. ^20–23^ for each lesion type. Normalized GSEA enrichment scores for signatures with at least one significant enrichment in any lesion type were plotted as a heatmap with pheatmap. Based on the GO BP analysis for BRL-specific genes and commonly expressed genes in all lesion types, genes with core enrichment for significant GO terms were combined to create signatures related to apoptosis regulation, cytokine pathways, immune activation, myeloid cell activation and migration, protein folding, transport and metabolism for the BRL-specific analysis, as well as adaptive immune function, antigen processing and presentation, B cells, innate immune function and T cells for the common gene analysis, respectively. GSEA plots were created for these GO-based group signatures with the Bioconductor package clusterProfiler; a ranked gene list based on the fold changes between BRL and NAWM expression was used as basis.

### PET studies

The PET studies were performed at Turku PET Centre between 2009 and 2022. All patients gave written informed consent, after which they underwent the MRI and TSPO PET scans, and neurological examinations. The Finnish MS registry^59^ and relevant patient records were used to obtain retrospective timelines for each patient’s EDSS progression. Patients were defined as progressed if the EDSS had increased by more than 1.5 if the EDSS at the time of imaging was 0; if the EDSS had increased by more than 1.0 if the imaging EDSS was 1–5.5; or if the EDSS has increased by more than 0.5 if the imaging EDSS was more than 6. Progression had to be confirmed after 6 months (observation periods as indicated). All reported data on all study participants were acquired according to study protocols previously approved by the Ethics Committee of the Hospital District of Southwest Finland. All study participants provided written informed consent according to the principles of the Declaration of Helsinki. Participants received no compensation. The sex of the participants was determined based on self-report.

### MRI and image processing

Most (*n* = 99) of the PET cohort underwent 3T MRI (Philips Ingenia/Philips Ingenuity) with the following sequences: fluid-attenuated inversion recovery, T2, three-dimensional (3D) T1, 3D gradient recalled echo (GRE) and DTI. Fifteen participants were imaged with 1.5T MRI (Philips Gyroscan Intera Nova Dual) with fluid-attenuated inversion recovery, T2 and 3D T1 sequences. T1 lesion masks were drawn and determined using manually checking and editing the lesion masks created with the Lesion Segmentation Toolbox in statistical parametric mapping (SPM12 software)^59,60^. The Lesion Segmentation Toolbox lesion filling tool was then used similarly as described in ref. ^61^ to create masks for the NAWM. FreeSurfer (v.7.3.0; surfer.nmr.mgh.harvard.edu/) was used to segment brain volumes, gray matter and thalamic volumes for the PET analyses and volumetric comparisons between groups. ExploreDTI v.4.8.6 running in SPM was used for DTI coregistration. DTI data were corrected for subject motion and geometric distortions, and tensor estimation was performed according to a method described previously^62^. The QSM images (*n* = 41) based on the 3D GRE sequence were processed as described previously^59^. QSM images were acquired using a 3D flow-compensated GRE sequence with 60 slices, 2-mm thickness, an acquisition matrix 240 × 187 mm (Ingenia) or 240 × 183 mm (Ingenuity), field of view = 240 × 184.8 mm (Ingenia) or 240 × 184 mm (Ingenuity), reconstructed voxels of 0.6 × 0.6 × 2 mm, FA = 15, echo time/repetition = 5.7/55 ms and AT = 3:49 (Ingenia) or 5:10 (Ingenuity) mm:ss. If the QSM signal was morphologically consistent with a lesion-associated ring, this lesion was determined as a QSM iron-rim lesion according to visual inspection by two experienced raters.

### [^11^C]PK11195 radioligand production and PET analysis

The [^11^C] radioisotope production and the [^11^C]PK11195 radiosynthesis followed a procedure described previously^59^. For each individual, 60 min of dynamic list mode PET data were acquired with a high-resolution research tomograph (Siemens Medical Solutions) with 1.22-mm slice thickness, and transaxial and axial spatial resolution of 2.5-mm full width at half maximum. Image reconstruction and post-processing followed previously described procedures to acquire realigned and resliced PET images to match the 1-mm voxel size of the MRI images^61^. Specific binding of [^11^C]PK11195 in the NAWM, measured as DVR, was quantified with the Logan method^63^ and time activity curves representing a cluster of voxels without specific binding, which were acquired with the MATLAB (MathWorks) software Super-PK^59^. Furthermore, parametric DVR maps were calculated from parametric binding potential maps (DVR = BP_ND_ + 1)^60^. Parametric DVR maps of each individual’s PET lesion masks were dilated in 2-mm steps and up to 4 mm in each direction; they were then subtracted from the previous iteration to obtain 0–2 mm and 2–4 mm perilesional masks. To determine a voxel-level threshold for an aberrantly high PET DVR, the mean DVR in the WM from 34 healthy controls (29% males, mean age = 46.7 years, that is, no significant difference with regard to age and sex compared to the MS cohort) was first calculated; +1 s.d. of this mean (DVR = 1.3624) was used to signify aberrantly HIGH activity, while areas with average values below this level were classified as LOW activity. Both 2-mm rims were then categorized as HIGH or LOW based on the average DVR within the rim. Lesions exhibiting a HIGH-HIGH pattern within both 2-mm segments were classified as BRLs. Additionally, each lesion was phenotyped as inactive, rim-active or overall active using the 0–2 mm perilesional area according to previously described methods: inactive lesions were defined as lesions with 0% active voxels at the core and rim; rim-active lesions were defined either if 5–20% active voxels were present in the core (rim must have at least twice the percentage of active voxels compared to the core) or if less than 5% active voxels were present in the core (rim must have at least 5% more active voxels than the core). Overall active lesions were those not fitting into either of the aforementioned categories^60^.

### Correlation of rBRLs with pathology

One case from the TSPO PET cohort, a male with progressive MS (aged 44.5 years with a disease duration of 15 years, with an EDSS of 7 at the time of PET imaging and treated with natalizumab between the ages of 33 and 35) came to autopsy after serial MRIs obtained 7.5, 6 and 2 years earlier. TSPO PET was performed 6 years before his death. His brain was attained at autopsy with informed consent provided by the next of kin (in accordance with Finnish legislation). MRI and gross pathology were matched. Two tissue blocks from one lesion with sufficient periplaque WM were available to determine the broadness of the microglial rim. Tissue sections were stained for HLA-DR and MBP (Extended Data Fig. 7).

### Statistical analysis and reproducibility

All staining was performed using an automated immunostainer (Autostainer Link 48). All staining conditions were tested using positive and negative controls. All tissue sections were stained once with the corresponding antibody. If the results of the staining were unsatisfactory (insufficient staining intensity or floating of tissue areas), the staining was repeated. If the staining results remained unsatisfactory, the individual section was excluded from the analysis. To compare the histological cohorts, all statistics were calculated using Prism v.10.2.1 (GraphPad Software). To compare two groups, an unpaired *t*-test or Mann–Whitney *U*-test was performed (depending on the normality of the variable checked with the Shapiro–Wilk test). To compare proportions, a Fisher’s exact test or chi-squared test was used. To compare three or more groups, the Brown–Forsythe test or Welch’s ANOVA for multiple comparisons, or the Kruskal–Wallis test with Dunn’s correction for multiple comparisons, was used.

Statistical analysis of the imaging data was performed using Prism (v.10.2.1). Groups were compared with an unpaired *t*-test or Mann–Whitney *U*-test (depending on the normality of the variable checked with the Shapiro–Wilk test); proportions were compared with a Fisher’s exact test. To compare three groups, a Kruskal–Wallis test with Bonferroni correction was used. Spearman rank correlation coefficients were used to evaluate the associations between continuous and ordinal variables.

Patients who reached an EDSS of 4 (*n* = 18) or 6 (*n* = 9) within 12 years from disease onset were categorized as ‘fast progressors’, while patients with an EDSS of less than 2 (*n* = 19) after 12 years were categorized as ‘slow progressors’. A subcohort was evaluated for clinical progression from PET imaging onwards according to EDSS change as published elsewhere^64^. All tests were two-tailed and *P* values were classified as significant if *P* < 0.05 (*P < 0.05, **P < 0.01, ***P < 0.001).

### Reporting summary

Further information on research design is available in the Nature Portfolio Reporting Summary linked to this article.

## Online content

Any methods, additional references, Nature Portfolio reporting summaries, source data, extended data, supplementary information, acknowledgements, peer review information; details of author contributions and competing interests; and statements of data and code availability are available at 10.1038/s41591-025-03625-7.

## Supplementary information

Reporting Summary

Supplementary Tables 1–8.

## Source data

Source Data Fig. 2 Cohort data and histology.

Source Data Fig. 3 Histology.

Source Data Fig. 4 Spatial transcriptomics.

Source Data Fig. 5 PET MRI imaging cohort and data.

Source Data Extended Data Fig. 3 Histology.

Source Data Extended Data Fig. 4 Histology.

Source Data Extended Data Fig. 5 Cohort data and SNP frequency.

Source Data Extended Data Fig. 6 Spatial transcriptomics.

Source Data Extended Data Fig. 8 PET MRI imaging and cohort.

## Data Availability

The spatial transcriptomics data files have been deposited at the Gene Expression Omnibus under the accession no. GSE281807. The data regarding the PET analysis are not publicly accessible due to the protection of patients’ privacy. Anonymized raw PET data are available over the next 3 years upon reasonable request from a qualified investigator, via the corresponding author. Requests will be addressed within 1 month from the request. Source data are provided with this paper.

## Code availability

The code used to analyze the study’s spatial transcriptomics data is available on GitHub (https://github.com/walter-ca/MS-lesions_code).
