# Supplementary material for: Broad rim lesions are a new pathological and imaging biomarker for rapid disease progression in multiple sclerosis
Source: Nat Med. 2025 Apr 29;31(6):2016–26. doi: 10.1038/s41591-025-03625-7 (PMC12176629; doi:10.1038/s41591-025-03625-7)
Supplement: Supplementary file 1 — Reporting Summary [file 41591_2025_3625_MOESM1_ESM.pdf]

nature portfolio

Corresponding author(s): Tanja Kuhlmann
Last updated by author(s): Feb 13, 2025

Reporting Summary

Nature Portfolio wishes to improve the reproducibility of the work that we publish. This form provides structure for consistency and transparency in reporting. For further information on Nature Portfolio policies, see our Editorial Policies and the Editorial Policy Checklist.

Statistics

For all statistical analyses, confirm that the following items are present in the figure legend, table legend, main text, or Methods section.

n/a	Confirmed	
☐	☒	The exact sample size (*n*) for each experimental group/condition, given as a discrete number and unit of measurement
☐	☒	A statement on whether measurements were taken from distinct samples or whether the same sample was measured repeatedly
☐	☒	The statistical test(s) used AND whether they are one- or two-sided<br>*Only common tests should be described solely by name; describe more complex techniques in the Methods section.*
☐	☒	A description of all covariates tested
☐	☒	A description of any assumptions or corrections, such as tests of normality and adjustment for multiple comparisons
☐	☒	A full description of the statistical parameters including central tendency (e.g. means) or other basic estimates (e.g. regression coefficient) AND variation (e.g. standard deviation) or associated estimates of uncertainty (e.g. confidence intervals)
☐	☒	For null hypothesis testing, the test statistic (e.g. *F*, *t*, *r*) with confidence intervals, effect sizes, degrees of freedom and *P* value noted<br>*Give P values as exact values whenever suitable.*
☒	☐	For Bayesian analysis, information on the choice of priors and Markov chain Monte Carlo settings
☒	☐	For hierarchical and complex designs, identification of the appropriate level for tests and full reporting of outcomes
☐	☒	Estimates of effect sizes (e.g. Cohen's *d*, Pearson's *r*), indicating how they were calculated

Our web collection on statistics for biologists contains articles on many of the points above.

Software and code

Policy information about availability of computer code

Data collection	Transcriptomic analyses: Raw fastq files were processed with the NanoString GeoMxNGSPipeline v2.3.3.10 on a Linux-based server system, and counts per gene were exported as NanoString DCC files per sample. The count files were imported into R v4.2.1 using the R/Bioconductor package GeomxTools v3,0.1 (GeomxTools: NanoString GeoMx Tools. R package version 3.0.1) and the Probe Kit Configuration (PKC) file Hs_R_NGS_WTA v1.0.<br>PET analysis: PET data was acquired with a high-resolution research tomograph (HRRT; Siemens Medical Solutions) PET scanner and MRI data with 3T MRI (Philips Ingenia/Philips Ingenuity) or 1.5 T MRI (Philips Gyroscan Intera Nova Dual) scanners. Raw MRI and PET data was exported from the data repository in dicom format and SPM12 running in Matlab R2017a was used to convert them to NIfTI. DTI data was acquired from the raw images with ExploreDTI v4.8.6 and the QSM data with Morphology Enabled Dipole Inversion (MEDI) toolbox with automatic uniform cerebrospinal fluid zero reference (MEDI+0).
Data analysis	Transcriptomic analyses: For the initial quality control, the minimum negative control counts were set to 1, while all other parameters were kept at their default values. Only samples with a gene detection rate > 0.03 were selected for further analyses. For the integrated analysis of the two NanoString runs, the R/Bioconductor package standR (v1.6.0) was employed to assess the performance of different batch effect removal strategies. Batch effects were corrected with standR's geomxBatchCorrection function, using RUV4 with k=5 and negative control genes based on the GeoMx data's slides. Normalization of data was performed using standR's geomxNorm normalization function with TMM as method, and dimensionality reduction was performed using principal component analysis (PCA). The R/Bioconductor pipeline limma v3.52.4 was employed to identify differentially expressed genes (DEGs) between the lesion rims and NAWM samples (FDR < 0.05), and between lesion types. DEGs were visualized with a ggplot2-based volcano plot (v3.4.4), and additional genes of interest with an FDR < 0.001 were marked in the volcano plot according to their level of significance. Gene set enrichment analyses (GSEA) were calculated with the R/ Bioconductor package clusterProfiler v4.7.112 and the Gene Ontology (GO) Biological Processes (BP) database (R/Bioconductor package org.Hs.eg.db, v3.15.0). Only gene sets with a minimum size of 10, a maximum size of 500 genes, and an adjusted p-value < 0.05 were

considered further. GO terms with significant enrichment were depicted as dotplots to show normalized enrichment scores (NES), adjusted p-values, and gene ratios indicating the percentage of genes with core enrichment for the respective gene sets.
Based on the DEG results between lesions and control, common lesion-related genes were defined as genes that were significantly upregulated for each of the three lesion types when compared to NAWM. Similarly, genes with a significant enrichment for only one lesion type were defined as lesion-specific genes for the respective lesion type. Overlaps of DEGs between lesion types were visualized as a Venn diagram with the R package VennDiagram v1.7.3. Furthermore, violin plots for chosen genes of interest were created with ggplot2, and a principal component analysis based on the normalized expression data for each sample was visualized with the same package. Based on the combined lists of DEGs between lesions, a filtered expression matrix was created, and pairwise Spearman correlation coefficients were calculated with the basic R cor function. The correlation matrix was visualized as a heatmap with pheatmap v1.0.12, using unsupervised clusterings for rows and columns based on pheatmap's default euclidean distance and the complete clustering method.

PET data analysis: GraphPadPrism 10.2.1 was used to analyze all PET data.

For manuscripts utilizing custom algorithms or software that are central to the research but not yet described in published literature, software must be made available to editors and reviewers. We strongly encourage code deposition in a community repository (e.g. GitHub). See the Nature Portfolio guidelines for submitting code & software for further information.

Data

Policy information about availability of data

All manuscripts must include a data availability statement. This statement should provide the following information, where applicable:

- Accession codes, unique identifiers, or web links for publicly available datasets
- A description of any restrictions on data availability
- For clinical datasets or third party data, please ensure that the statement adheres to our policy

Spatial transcriptomics data files are deposited at NCBI GEO under the accession number GSE281807 which was made public. The R/Bioconductor package org.Hs.eg.db, v3.15.0 was used as database for Gene Ontology (GO) Biological Process (BP) information.

The data regarding PET analysis are not publicly accessible due to the protection of patients' privacy. Anonymized raw PET data is available over the next 3 years on a request from a qualified investigator, via the corresponding author. Requests will be addressed within one month from the request.

Human research participants

Policy information about studies involving human research participants and Sex and Gender in Research.

Reporting on sex and gender	The histology cohort consisted of 30% males and 70% females.<br>The TSPO-PET cohort consisted of 28 % males and 72 % females.
Population characteristics	The autopsy cohort consisted of cases with a clinical and pathologically-confirmed diagnosis of multiple sclerosis, with a mean age of 62 years (range 32-95) at autopsy. In the severe sub-group, 35% wash an immune-therapy in the course of disease, in the benign group this was 6%).
Recruitment	Participants are included in the Netherlands Brainbank donor-program, which provides a sub-selection for participants according to the Dutch demographic buildup with willingness to donate brain material postmortem. This may limit the applicability of our findings on the general MS population.<br>TSPO-PET patients were recruited by the treating neurologists from the Turku University Hospital neurology clinic between 2009-2022. All patients with confirmed MS disease (aged 20 -70) with willingness to participate in a PET imaging study were considered eligible for inclusion. All patients with simultaneous PET and MRI were included in this study, regardless of treatment, clinical status or sex/gender.
Ethics oversight	We used archival samples. The research upon anonymization was in accordance with local ethical standards and regulations by the Ethics Committee of VU University Medical Center, Amsterdam, The Netherlands, the Ethics Committee of the Hospital District of Southwest Finland and the Ethics Committee of the University Münster.<br>PET data: All study subjects in PET analyses were acquired according to study protocols previously approved by the Ethics Committee of the Hospital District of Southwest Finland. All study participants provided written informed consent according to the principles of the Declaration of Helsinki.

Note that full information on the approval of the study protocol must also be provided in the manuscript.

Field-specific reporting

Please select the one below that is the best fit for your research. If you are not sure, read the appropriate sections before making your selection.

☒ Life sciences ☐ Behavioural & social sciences ☐ Ecological, evolutionary & environmental sciences

For a reference copy of the document with all sections, see nature.com/documents/nr-reporting-summary-flat.pdf

Life sciences study design

All studies must disclose on these points even when the disclosure is negative.

Sample size	Patient samples were stratified based patient narratives, along with dates and ages of registration of functional equivalents of EDSS score. All available tissue blocks per selected patient were screened for lesions.<br>PET-data: all available subjects with successful PET and MRI imaging in the Airas Group study cohorts imaged at Turku PET Centre were included.
Data exclusions	For the transcriptomic analysis, areas of illumation (AOIs) with a gene detection rate of less than 3% were excluded from the analysis. AOIs with a gene detection rate of 3% or higher showed expected marker expressions and a consistent clustering with clear separation from another cohort of AOIs, while AOIs with a gene detection rate of less than 3% were excluded for low gene expression signal and unspecific signal patterns. This threshold was not pre-established.<br>For histology and PET studies, no data were excluded.
Replication	For histological quantification at least ten visual fields per region of interest were analysedand all were successfully included in the analysis.<br>Transcriptomics: A total of 269 areas of illumination were measured, originating from 28 different patients (list of replicate numbers and lesion types is included as Supplementary Table 1). After filtering, 212 areas of illumination were used for further analyses.
Randomization	For histology and transcriptomic analysis, the samples were allocated in random experimental groups. For PET study, no groups were allocated.
Blinding	Samples were selected according to the patients disease course.<br>For histological analysis the subtypes are directly detectable and blinding is therefore not possible. For PET study, no groups were allocated.

Reporting for specific materials, systems and methods

We require information from authors about some types of materials, experimental systems and methods used in many studies. Here, indicate whether each material, system or method listed is relevant to your study. If you are not sure if a list item applies to your research, read the appropriate section before selecting a response.

Materials & experimental systems

n/a	Involved in the study	
☐	☒	Antibodies
☒	☐	Eukaryotic cell lines
☒	☐	Palaeontology and archaeology
☒	☐	Animals and other organisms
☒	☐	Clinical data
☒	☐	Dual use research of concern

Methods

n/a	Involved in the study	
☒	☐	ChIP-seq
☒	☐	Flow cytometry
☐	☒	MRI-based neuroimaging

Antibodies

Antibodies used	CD163 (Leica NCL-L-CD163 Lot.:6063445)<br>CD206 (Sigma HPA004114 Lot.: 46663)<br>CD3 (Dako M7254 Lot.: 4141595)<br>CD68 (Dako M0814 Lot.: 20040389)<br>CD74 (Sigma HPA010592, Lot.: C113825)<br>CD79a (Dako M7050 Lot.: 20043883)<br>CHIT1 (Sigma HPA010575, Lot.: 27635)<br>GFAP (Dako Z0334 Lot.: 20035994)<br>HLA-Dr (Dako M077501-2 Lot.: 41337748)<br>Iba I (Wako 019-19741 Lot.: SKN4887)<br>iNos (Sigma ABN26 Lot.: 3928023)<br>MBP (Abcam ab7349 Lot.: 1015269-34)<br>PLP (Invitrogen, #PA5-102820, Lot.: ZG4381884A)<br>TEMEM 119 (Sigma HPA051870 Lot.: 41615)<br>TPPP (Abcam ab92305 Lot.: GR3330149-4)<br>TSPO (Invitrogen MA5-33203 Lot.: ZI4454572)<br>CD68 (Santa Cruz sc-20060AF594 Lot.: #H1522)<br>OLIG2 (Abcam ab225100)<br>SYTO83 (Thermo Fisher S11364 Lot.: 2541524)<br>TPPP (Abcam ab204011 Lot.: 10362775)
Validation	Nonspecific background signals were assessed for all antibodies by omitting the secondary antibody. Additionally, the staining patterns for all antibodies were evaluated by appropriate cell-type-specific negative controls, such as neurons being negative for

astrocytic markers.

Magnetic resonance imaging

Experimental design

Design type: N/A, instructions of these questions were fMRI related. In this study fMRI was not used.

Design specifications: N/A, instructions of these questions were fMRI related. In this study fMRI was not used.

Behavioral performance measures: N/A, instructions of these questions were fMRI related. In this study fMRI was not used.

Acquisition

Imaging type(s): Structural (volumetric), quantitative susceptibility mapping (QSM; qualitative analysis), diffusion tensor (quantitative)

Field strength: 3T or 1,5 T

Sequence & imaging parameters: FLAIR, T2, 3DT1, 3D GRE and DTI

Area of acquisition: Whole brain

Diffusion MRI ☒ Used ☐ Not used

Parameters: 33 (n=17), 64 (n=48), or 67 gradient directions (n=17) with b value = 1,000 s/mm^2

Preprocessing

Preprocessing software: SPM8 or SPM12 running in MATLAB used for co-registration. Lesion Segmentation Toolbox (LST) in SPM12 used for preliminary lesion mask and for lesion filling. FreeSurfer 7.3.0 used to segment brain. ExploreDTI v4.8.6 running in MATLAB used for DTI data and MEDI toolbox for QSM data.

Normalization: T1 was co-registered to MNI space and then all other images were coregistered to T1 space.

Normalization template: MNI152 ICBM 2009a Nonlinear Symmetric (T1w)

Noise and artifact removal: Raw DTI data was corrected for subject motion, EPI/susceptibility and eddy current induced geometric distortions according to instructions using RESTORE tensor estimation method

Volume censoring: N/A, instructions of these questions were fMRI related. In this study fMRI was not used.

Statistical modeling & inference

Model type and settings: Volumetric, diffusion tensor and QSM data: T-tests or Mann-Whitney's U-tests, Fisher's exact tests and Kruskal-Wallis tests (with Bonfferoni correction) as well as Spearman rank correlation coefficients were used.

Effect(s) tested: N/A, instructions of these questions were fMRI related. In this study fMRI was not used.

Specify type of analysis: ☐ Whole brain ☐ ROI-based ☒ Both

Anatomical location(s): Lesion masks were drawn by manually checking and editing the masks created with the Lesion Segmentation Toolbox (LST) in SPM12. Freesurfer 7.3.0 was used for brain segmentation.

Statistic type for inference (See Eklund et al. 2016): N/A, instructions of these questions were fMRI related. In this study fMRI was not used.

Correction: N/A, no MRI analyses needed multiple comparison corrections.

Models & analysis

n/a	Involved in the study
☒	☐ Functional and/or effective connectivity
☒	☐ Graph analysis
☒	☐ Multivariate modeling or predictive analysis
